# Does Population Diversity Matter for Economic Development in the Very Long Term? Historic Migration, Diversity and County Wealth in the US

**DOI:** 10.1007/s10680-018-9507-z

**Published:** 2018-12-04

**Authors:** Andrés Rodríguez-Pose, Viola von Berlepsch

**Affiliations:** London School of Economics, Houghton St, London, WC2A 2AE UK

**Keywords:** Diversity, Fractionalization, Polarization, Economic development, Counties, USA

## Abstract

Does population diversity matter for economic development in the long run? Is there a different impact of diversity across time? This paper traces the short-, medium- and long-term economic impact of population diversity resulting from the big migration waves of the late nineteenth and early twentieth centuries to the United States (US). Using census data from 1880, 1900 and 1910, the settlement pattern of migrants across the counties of the 48 US continental states is tracked in order to construct measures of population fractionalization and polarization at county level. Factors which may have influenced both the individual settlement decision at the time of migration and county-level economic development in recent years are controlled for. The results of the analysis show that high levels of population fractionalization have a strong and positive influence on economic development in the short, medium and long run. High levels of polarization, by contrast, undermine development. Despite a stronger effect on income levels in the first 30 years, we find these relationships to be extremely long lasting: counties with a more heterogeneous population composition over 130 years ago are significantly richer today, whereas counties that were strongly polarized at the time of the migration waves have endured persistent negative economic effects.

**Electronic supplementary material:**

The online version of this article (10.1007/s10680-018-9507-z) contains supplementary material, which is available to authorized users.

## Introduction

In 2015, migration stock numbers worldwide exceeded expectations and rose to 244 million (UNDESA 2016). Growing cross-border population flows have contributed to a shift both in the political discourse and in the scientific research agenda, bringing the analysis of the economic implications of migration into the fore.

Over the past decades, a vast amount of new scientific research has led to considerable progress in our understanding of the economic implications of migration. The economic impact of migrants on both their own futures and that of locals (i.e. Borjas 1994; Card 2005), on the local labour market and its dynamics (i.e. Altonji and Card 1991; Friedberg and Hunt 1995; Bijak et al. 2007), and on public finances (i.e. Kerr and Kerr 2011) has been extensively analysed. Transmission channels—such as increasing returns to scale (i.e. Borjas 1995), alterations to the ratio of skilled to unskilled labour (Lundborg and Segerström 2002), wages (Ottaviano and Peri 2006), or the stimulation of productivity by means of innovation and specialization (i.e. Gordon and McCann 2005; Partridge and Furtan 2008)—have also been an object of greater scrutiny. The focus of these studies, however, has generally been short term. Our understanding of the economic implications of migration has commonly been limited to the first 5 to 10 years after the initial migration wave took place. The medium- to long-term impact of migration on economic prosperity has been mostly neglected. Only in recent years have researchers started to address this gap. In particular, recent work by Rodríguez-Pose and von Berlepsch (2014) has demonstrated how current levels of economic development across the US still depend on migration settlement patterns that took place over 100 years ago. Sequeira et al. (2018) have confirmed the significance of this relationship. This long-term impact of migration holds in time regardless of the national origin of migrants settling in different territories (Rodríguez-Pose and von Berlepsch 2015).

However, one important demographic aspect related to migration has remained firmly anchored in short-term scrutiny: diversity. As formerly homogeneous communities become more diverse by accommodating new individuals bringing their customs, traditions, ideas, abilities and experiences with them, the question of whether more diverse societies facilitate or deter growth has become more prominent. Research on the economic impact of population diversity has flourished, focusing on a multitude of transmission channels ranging from skill variety, social interaction, innovative networks, institutions and the provision of public goods to trust, social participation, social unrest and conflict (i.e. Easterly and Levine 1997; Alesina and La Ferrara 2005; Ottaviano and Peri 2006; Gören 2014; Alesina et al. 2016; Bove and Elia 2017; Kemeny and Cooke 2017). Most of this research has unveiled a considerable effect of diversity on growth over the short term. Yet, our knowledge about whether population diversity levels generated by past migration waves still affect economic outcomes over the medium and long term remains untouched within the scientific literature. In this respect, by looking at the impact of population diversity at county level more than one century ago on current levels of development, this paper fills a gap in our knowledge that has not been covered before.^1^

We seek to ascertain whether territories that were characterized by a large degree of population diversity more than a century ago are wealthier today compared to those that remained more homogenous in their population composition. Does having a very diverse population at one point in time lead to persistently higher levels of economic growth? Or is the economic impact of diversity only evident in the short term, vanishing once the different population groups become part of the society’s “melting pot”?

In this paper, we assess the extent to which the high degree of cultural diversity in US counties generated during the Era of Mass Migration of the late nineteenth and early twentieth century has left an enduring impact on the economic development of those areas of the US that witnessed the greatest heterogeneity in population. Incorporating a twofold definition of the notion of diversity, encompassing two distinct dimensions of the term (fractionalization and polarization), we undertake a decade-by-decade analysis for the US at the subnational level covering the period between 1880 and 2010. We posit that a vibrant, highly diverse population, stemming from a multitude of different backgrounds, nationalities and cultures has the capacity to leave a long-lasting economic impact. We argue that population diversity becomes embedded not only in local institutions but in the very core of a territory, affecting the subsequent economic development path of the region not only over the short term, but also over the medium and long term.

In order to test whether this is the case, the paper adopts the following structure: Section 2 gives an overview of the historical background of the Age of Mass Migration. Section 3 summarizes previous approaches to the link between diversity and economic development in the literature. In Sect. 4, we describe the model, methodological aspects and the data adopted for our research. We also explain the calculations of the various indices used in the paper as main variables of interest. Section 5 reports the results of our estimation, while Sect. 6 concludes.

## Mass Migration to and Within America: A Short Overview

When speaking of the Age of Mass Migration to the US, historians refer to the period between the pre-Civil War years and the mid-1920s. Within this time span, more than 40 million Europeans left their homelands affected to different degrees by political disturbances, famine and religious persecution in search of a new and better life. The large majority of these migrants chose the US as their final destination (Hatton and Williamson 1994; Bertocchi and Strozzi 2006). With an average annual immigration inflow rate of about 0.75% of the total US population at the time (Hatton and Williamson 1998), the US experienced a population increase in an extent which had been unheard of in contemporary history.^2^ During this period, the total US population increased sixfold, from about 17 to 105 million. Meanwhile, the proportion of the foreign-born white population grew from 13% in 1850 to approximately 18% in 1910 (Table 1). Most importantly, “the proportion of people of foreign birth and parentage together reached its maximum level of 45% in 1920” (Ward 1990: 299).

**Table 1:** US population composition, 1840–1920 (in percentages of total population)

Year	Population (millions)	Black (%)	Foreign parentage (%)	Foreign born (%)
1840	17.1	16.8	n.d.	n.d.
1850	23.2	15.7	n.d.	12.9
1860	31.4	14.1	n.d.	17.9
1870	39.8	13.5	19.0	19.6
1880	50.2	13.1	22.5	17.8
1890	62.9	11.9	25.0	19.9
1900	76.0	11.6	27.6	18.1
1910	92.0	10.7	27.8	18.0
1920	105.7	9.9	28.0	16.9

*Source*: Ward (1990)

*n.d.* no data

At that time, no restricting legislation prevented incoming migrants from entering the country. Migrants—no matter which nationality—could roam freely and settle wherever they wished.^3^ The introduction of the literacy test in the Immigration Act of 1917 led to the first serious restriction to immigration. Quotas for incoming migrants followed in 1921 (Emergency Quota Act of 1921). By 1924, entry restrictions for all foreigners were passed (Goldin 1994; Alexander 2007).

**Fig. 1:**
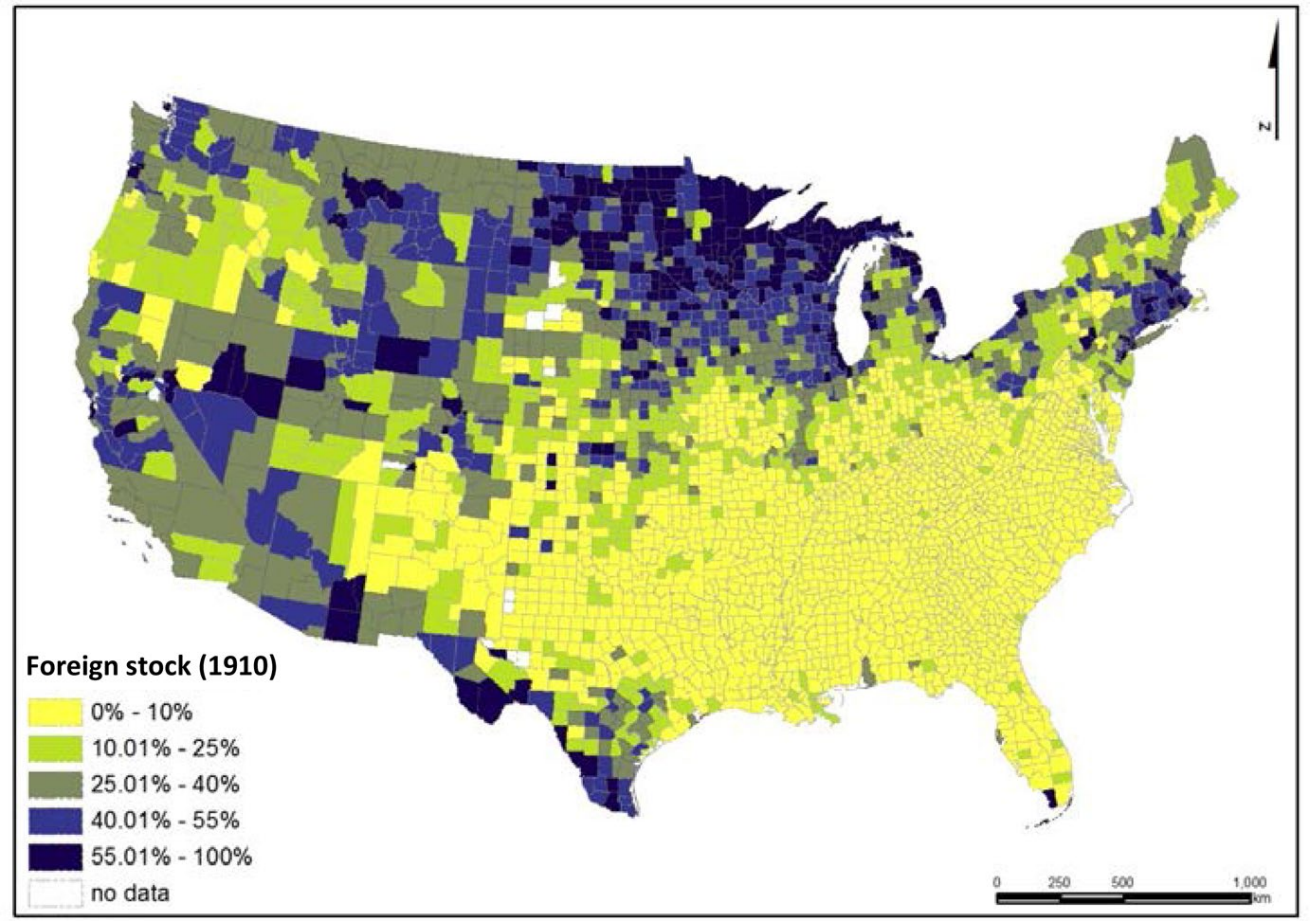
International migrants and their children as share of population by county in 1910. *Source*: Rodríguez-Pose and von Berlepsch (2014)

Most newcomers settled where relatives and friends had already settled (e.g. Vedder and Gallaway 1972; Levy and Wadycki 1973; Dentlevy and Gemery 1977) creating distinct migrant communities across the country. Hence, regions with large migrant networks attracted further newcomers, while others remained nearly untouched by this mass movement of population. The resulting settlement pattern is depicted in Fig. 1 for 1910.^4^

The North and West of the country attracted most migrants. Southern states remained, by contrast, largely inhabited by American-born residents. Migrants established themselves in the rural areas of Illinois, Iowa, Michigan, Minnesota and Wisconsin (Fig. 1) and further to the west in sparsely populated counties, as well as in southern Texas and the southern tip of Florida in the South. Cities, especially in New England and the Atlantic states, as well as Chicago became big magnets for migrants, especially for those entering the country during the second migration wave between 1890 and 1920.

While international migration rose rapidly, changing the population composition of large parts of the United States, internal migration also picked up speed, reaching exceptionally high geographical population mobility levels. At the end of the nineteenth century, almost 60% of the male US population above the age of 30 had moved across county or state lines and almost a third of those born in the US lived outside their place of birth (Haines 2000; Ferrie 2005). Similar to international migrants, American-born population moved especially westward in search of land to expand the wheat, corn, wool and meat production (Atack et al. 2000).

**Fig. 2:**
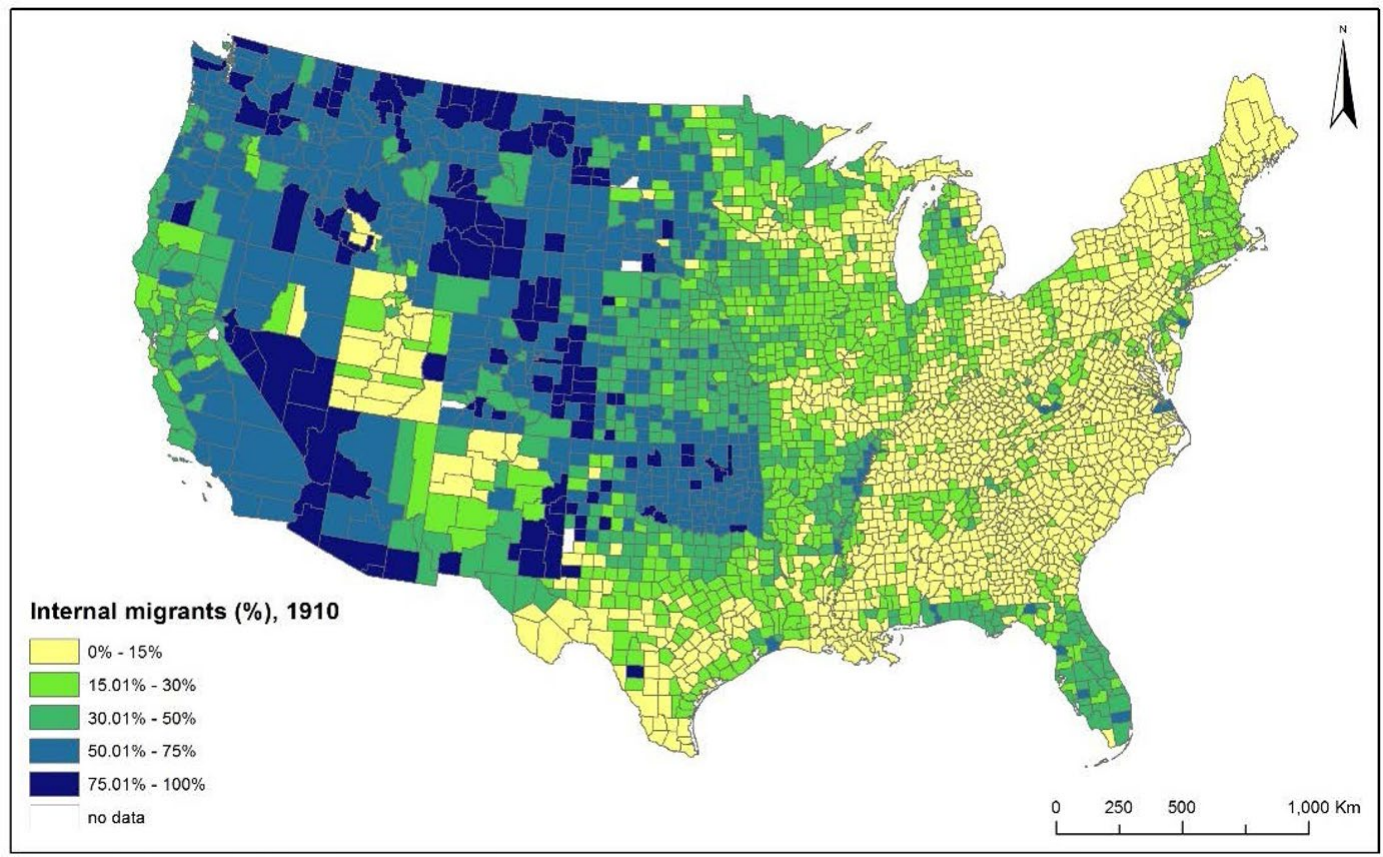
American-born internal migrants as share of population by county in 1910. *Source*: Own elaboration, using Ruggles et al. (2015) data

Most internal migrants of the late nineteenth century, however, travelled only over short distances, with the majority remaining within their state of birth. Twenty percent, however, covered much larger distances, in some cases up to 4500 km (own calculations). Figure 2 depicts their settlement pattern in 1910. The resulting map reveals a different geography of American-born internal migration than that of international migration. Internal migrants mainly moved from East to West, settling in many states west of the Mississippi (with the exception of Utah, New Mexico, Texas and parts of California). The majority of the population of midwestern states such as Oklahoma, Wyoming, Montana, Oregon, Nevada and Arizona was thus made up of internal migrants. The entire eastern and southern parts of the country (including the growing migrant agglomerations in the eastern cities) remained, with the exception of Florida, outside of internal migration routes.

Both international and internal migration movements affected the population composition of the US drastically. In parts of the north-western states, for example, within a few years the population changed from being almost entirely local-born to rates of 90% or more born in other US states or abroad. Internal migrants originating from locations often thousands of kilometres away were as foreign to the local population as the Germans, Irish, Italians or Poles settling within the same county. Whilst their language was the same—as was the case for migrants from the British Isles—internal migrants brought habits, customs, traditions and a life style which was regarded as outlandish and strange by the local population (Merk 1978).

**Fig. 3:**
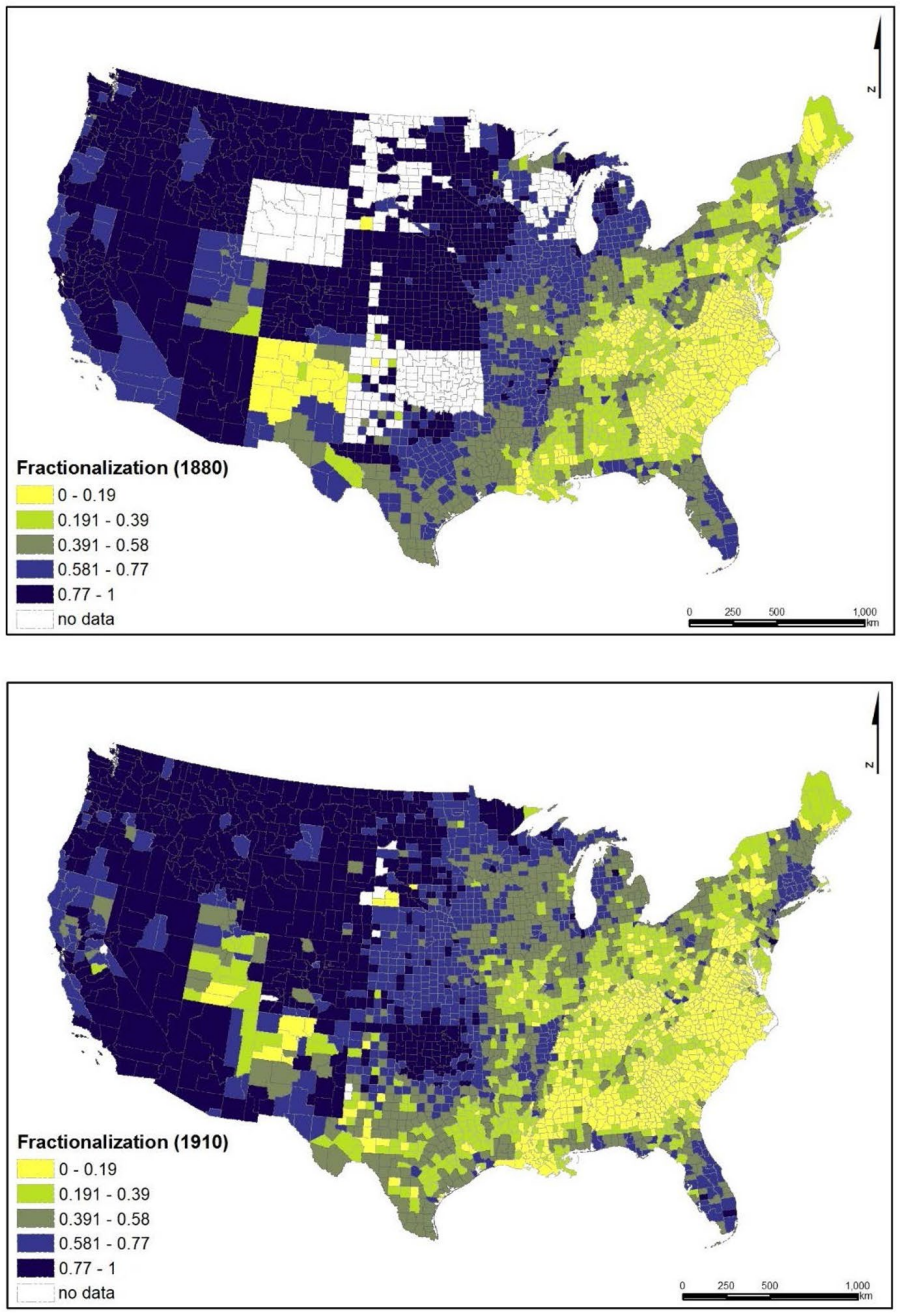
Diversity in the composition of the population by county in 1880 and 1910. *Source*: Own elaboration, using Ruggles et al. (2015) data

Whereas some areas of the US were predominantly settled by one or two specific nationalities (Rodríguez-Pose and von Berlepsch 2015), other regions attracted a multitude of migrants stemming from all over the United States as well as from a variety of different countries, leading to high levels of population diversity. Figure 3 displays the levels of population diversity—proxied by the widely used index of fractionalization, which emphasizes the number of different groups within a population—across US counties in both 1880 and 1910.^5^ High levels of population diversity became the norm primarily in the West of the country (with the exception of parts of New Mexico, Texas and Utah), while huge swaths of the old South remained demographically homogeneous. Cities in the North East, such as New York City and Boston, hosted vibrant, mixed migrant communities. By contrast, other areas in the North East, such as Maine, Vermont, or parts of upstate New York, were characterized by low population diversity levels generally ranging between 0 and 0.3.

## Diversity and Economic Development

With both international and internal migrants arriving and moving around, population diversity across the US changed drastically (Collier 2013). How such a rapid shock to diversity levels has affected ensuing economic development is therefore a highly relevant question. Whether population diversity leads to higher or lower growth has turned into a widely discussed and often controversial topic in the theoretical and empirical literature across a wide range of disciplines, ranging from sociology and anthropology to political science, demography, geography, and economics. Overall, conclusions are far from clear-cut due to a mix of alternative indices, changing geographical units and varying aggregation levels.

Two opposing strands dominate the debate—one depicting diversity as growth-enhancing, the other as growth-reducing. As the definition of diversity is far from straightforward, the strongly differing views result primarily from the respective dimension of diversity examined. Both strands choose entirely different angles to evaluate the link between diversity and economic development. Consequently, a variety of indices as proxy of diversity is used, with each indicator measuring a different aspect of the notion. The most popular proxies used are measures of population fractionalization, on the one hand, and polarization and segregation, on the other. Hence, whether diversity fosters or deters growth strongly hinges on the indicator employed.

The strand of research considering diversity as growth-enhancing generally regards it as a central driver of innovation and creativity, which in turn fosters technological progress and growth. Migrants arriving from diverse locations are depicted as an important input in the process of technological progress. They bring different skills, ideas, experiences and abilities to their places of destination. However, the speed of technological progress fuelled by the inflowing population does not depend on the size of the influx but on their composition, transforming diversity into a productivity-enhancing and innovation-initiating factor (Bove and Elia 2017).

The connection between diversity and innovation dates back at least to Jacobs (1961, 1969). For her, environments which are characterized by the presence of a large variety of cultural groups provide a more fertile soil for new ideas. Within these idea-breeding grounds, new innovative concepts can spread more easily to different areas than in more homogenous places, thereby fostering innovation and growth. Florida’s creative class model (2002) supports this line of argumentation. As skilled, liberal people prefer to live in diverse regions, skilled jobs and innovation will cluster in these same areas. The “New Argonauts” theory developed by Saxenian (2006) is yet another example of diversity leading to innovation. The concept revolves around foreign-born, technically high-skilled entrepreneurs, travelling back and forth between their home countries and the Silicon Valley, boosting economic activity both in the once-peripheral regions of their home countries, and in the United States. Lazear (1999) draws a parallel to a firm context analysing the globalization of firms. He finds that skill complementarity in a team spanning multiple cultures is key to not only offset the potential costs of diversity, but to significantly raise overall firm productivity. The interaction of a multitude of people with different abilities, ideas and experiences triggers innovation, technological process, and hence growth.

Empirical research has tended to validate this view. Niebuhr (2010) shows that patent applications increase in proportion to labour force diversity across German regions. Özgen et al. (2011a) find that innovation levels rise with the degree of diversity in the migrant community across European countries. A diverse labour force and immigrants originating from a wide range of countries “not only contribute to innovation by means of their high skills and innate abilities, but […] they also bring into firms and host countries new ideas and perspectives from their different cultural backgrounds” (Özgen et al. 2011b: 1). An enlarged national-origin pool is also linked to improvements in problem-solving, new combinations of ideas and innovation (i.e. Hong and Page 2001, 2004), while interethnic ties contribute to increase the socio-economic status of migrants (Riedel 2015). Alesina et al. (2016) report that the productive effects of increasing population diversity are largest for high-skilled migrants and for migrants stemming from wealthier and more culturally similar source countries.

Ottaviano and Peri (2006) unveil a significant positive and robust correlation of both wages and rents with regional immigrant diversity in US metropolitan areas, emphasizing that a more multicultural environment increases the productivity level of US-born citizens. From a slightly different angle, other studies portray diversity as productivity-enhancing not only in regions or cities but also in work establishments. The enlarged skill set of the workforce as well as the interaction of these diverse work teams with each other facilitates the production of a larger variety of goods and services, raising labour productivity levels, even when holding average ability constant (i.e. Alesina and La Ferrara 2005; Hamilton et al. 2003; Trax et al. 2015; Kemeny and Cooke 2017).

Two common denominators link the above diversity-promoting studies. Firstly, the large majority places the emphasis on the subnational, more granular level, evaluating either the impact of diversity at a regional, city, or even individual level. Secondly, and more importantly, this strand of research generally considers diversity on the basis of the number of different population groups—varying by language, religion or ethnicity—within a territory. They tend to use an index of fractionalization—such as Alesina et al.’s (2003)—as the measure of population diversity. This type of index presupposes that the greater the number of groups, the higher the assumed diversity in a society, positively influencing the potential for growth. The groups’ size or the distance between them does not enter the calculation of the most frequently used indices.

The strand of research positing that diversity has a negative influence on economic development follows a different line of thought. Rather than focusing on the positive influence of diversity on idea generation, innovation and productivity, it considers the presence of diverse groups as destabilizing factor within a society enhancing the potential for social unrest and conflict. This group not only takes into account fractionalization as proxy for diversity but increasingly refers to the use of indices of segregation and polarization.

“When the society is divided by religious, ethnolinguistic or race differences, tensions emerge along these divisions” (Montalvo and Reynal-Querol 2005a: 308). Ethnolinguistic fractionalization has thus been inversely linked to per capita GDP and long-run growth in large cross-country samples (e.g. Easterly and Levine 1997; Alesina et al. 2003; Churchill and Smyth 2017). Alesina et al. (2003), for example, find that the difference between a wholly homogenous and a wholly heterogeneous society represented up to 1.9 percentage points in economic growth in favour of the former. The poor economic performance of African countries has been, in particular, blamed on ethnic conflict, resulting from high levels of national or ethnic polarization (Easterly and Levine 1997).

Various channels have been identified as vehicles through which diversity hinders economic development. Gören (2014) emphasizes the negative direct effect of diversity for economic growth and considers polarization an indirect source of negative economic effects via human capital, investment, openness and civil war. Easterly and Levine (1997) point to a reduced probability of adopting “good policies” in more polarized societies. According to their study, low school attainment, financial debt and low infrastructure quality are all consequences of high segregation levels. Moreover, diversity is believed to foster rent-seeking behaviour by different groups, further undermining the potential for adopting sound public policies. Overall, high polarization triggers “positive incentives for growth-reducing policies, such as financial repression and overvalued exchange rates, that create rents for the groups in power at the expense of society at large” (Easterly and Levine 1997: 1206).

More fragmented societies are found to curb public sector performance and to generate poor institutions (La Porta et al. 1999; Mauro 1995; Easterly et al. 2006), leading to regional disparities (Ezcurra and Rodríguez-Pose 2013, 2017), an inefficient provision of public goods and services, a reduction in government transfers and distortionary taxation (Desmet et al. 2009; Azzimonti 2011), political instability (Alesina et al. 1999, 2003; Baldwin and Huber 2010), as well as increasing property rights insecurity (Keefer and Knack 2002) and low quality of government (Alesina and Zhuravskaya 2011). Enhanced heterogeneity may even result in the formation of xenophobic political parties (ibid), undermine collective action and reduce the efficiency of regulation (Baland and Platteau 2003; Platteau and Seki 2007).

Diversity is further shown to impact political rights, adversely affecting economic growth (Collier 2001). Particularly in less democratic societies, polarization can curtail individual rights and limit overall economic performance (Bluedorn 2001; Alesina et al. 2003). Further consequences of highly diverse and polarized societies are a reduction in trust and social participation, inefficient communication, less economic integration, lower voting turnout and a rise in transaction costs for bridging cultural differences (i.e. Ancona and Caldwell 1992; Alesina et al. 1999; Alesina and La Ferrara 2000; Richard et al. 2002; Van Knippenberg and Schippers 2007; Alesina and Zhuravskaya 2011; Uslaner 2011; Mavridis 2015; Martinez i Coma and Nai 2017). The resulting rent-seeking behaviour leads to slower growth, lower production, reduced investment and diminished prosperity (Rodrik 1999; Alesina and La Ferrara 2005, Montalvo and Reynal-Querol 2005b). “In extreme cases, diversity can prompt large-scale social and economic collapse, sometimes with horrific consequences, as has occurred in recent years in parts of Central Africa, the Balkans, and elsewhere” (Kemeny 2012). Highly fragmented societies have been deemed prone to moderate-intensity conflict. In highly polarized societies, conflict can be less frequent but of higher intensity (Esteban and Ray 2008). The likelihood and frequency of civil wars—an extreme example of social collapse—have been associated with high population diversity in terms of polarization (i.e. Horowitz 1985; Elbadawi and Sambanis 2002; Montalvo and Reynal-Querol 2005a, b).

Focusing on regional data, these results have been upheld by a number of studies analysing the case of the US. High diversity in US communities has been connected to a less efficient provision of public goods, lower trust and less social participation (i.e. Alesina et al. 1999; Alesina and La Ferrara 2000; Luttmer 2001; Alesina and La Ferrara 2002). Diversity has also been considered a strong and persisting barrier to developing trust across racial, ethnic or national origins (Glaeser et al. 2000).

Again, a string of common denominators links the above studies. Firstly, within this strand of the literature and with few exceptions, studies tend to use nations as the unit of analysis. Secondly, diversity is increasingly referred to as triggering the negative effects of polarization and segregation. Different indices have been employed by the literature in order to capture this effect. One of the most commonly used, proposed by Esteban and Ray (1994), finds its roots in the social tension literature. Here, indices measure entirely different aspects of diversity than fractionalization. Rather than focusing on the number of groups within a population, polarization indices emphasize their relative size to one another and the distance separating them. The bigger the distance among groups, the more similar their size, and the stronger the lines separating them the smaller the capacity to communicate and hence the larger the negative impact of diversity on economic development. According to Montalvo and Reynal-Querol (2005a), social unrest is further aggravated if the population is distributed into two separate groups of similar size.

In short, cultural diversity affects trust among the inhabitants of primarily multinational, multi-ethnic and multi-religious countries. It upsets the coordination of actors and their communication, generating animosity, enlarging differences in preferences and creating situations of conflict. Simultaneously, however, this multitude of experiences, skills and abilities can foster technological innovation, create a fertile soil for new ideas, increase productivity levels and therefore enhance the supply and the quality of goods and services. By influencing both human capital and the process of technological progress, diversity has an undeniable impact on economic growth with its net effect, however, remaining still unclear (Bove and Elia 2017).

One aspect which has been largely neglected in all of the aforementioned literature still needs to be evaluated: the dynamic economic impact of diversity over time. While there is big controversy about how fractionalization and/or polarization matter for economic growth, to the best of our knowledge, we know only very little about whether higher or lower initial levels of diversity—regardless of measurement—affect growth differently in the short, medium or very long term.

Research that dwells on the time dimension of diversity is few and far between. Campos and Kuzeyev (2007) or Campos et al. (2011) treat changing levels of polarization over time and examine their short-term impact on growth. Both find a negative effect of polarization. Alesina et al. (2016) and Ager and Brückner (2013a) consider time frames of 10 and 50 years, respectively, evaluating a sample of 120 countries with panel data between 1990 and 2000 (the former) or following a within-county estimation approach for US counties that measures the impact of the change in the cultural composition across a 50-year (1870–1920) period on economic growth (the latter). Both find fractionalization to be positively related to economic prosperity, while polarization has the opposite effect. However, neither assesses the impact of a fixed initial level of diversity on economic performance across alternating time horizons. Furthermore, while Ager and Brückner (2013a) base their study on the same historical time frame as used in this paper, they do not extend their analysis to present levels of economic development.

The studies that come closest to analyse a dynamic effect of diversity over longer time horizons are rare. They include Ager and Brückner (2013b) and Bove and Elia (2017). The former report a significant short- and long-term impact of initial diversity levels on economic development in the US. However, they refer to the use of genetic diversity based on Ashraf and Galor (2013), rather than including the two most frequently discussed proxies of diversity: fractionalization and polarization. Bove and Elia (2017) identify a positive association of both fractionalization and polarization with real GDP per capita, when evaluating a 135-country sample over a 50-year time frame. The positive link of both indicators—consecutively added to their model—is significant over the long term, but fails to retrieve consistent results over the short, 10- to 20-year time frame.

In short, to the best of our knowledge, there is no scientific research treating both dimensions of diversity—fractionalization and polarization—connecting historical population diversity levels to current economic development and covering a period longer than 50 years. Thus, some key questions remain unanswered: does diversity, in its two fundamental dimensions of fractionalization and polarization, shape growth—if at all—differently in the short term than in the medium and long term? Does a high degree of fractionalization and/or polarization generated more than a century ago promote growth in the short run, but limit it in the long term? Or is it vice versa?

## Empirical Approach

The aim of this paper is precisely to fill this important gap in the literature by examining the extent to which the levels of initial diversity, defined by both fractionalization and polarization, generated during the Age of Mass Migration across US counties: (a) have left a long-lasting economic legacy that can still be identified in the economic development of US counties today and (b) whether any positive or negative influence of initial diversity on economic development has waxed or waned with time.

Based on the previous discussion, we adopt two econometric models in order to test our two research questions: one focusing on population heterogeneity, the other on population homogeneity. Following the relevant literature, we employ place-of-origin fractionalization and polarization—the two most commonly used indices—to depict population diversity in Model 1. Its almost polar opposite, place-of-origin concentration, is used to reflect population homogeneity in Model 2.

We expect both dimensions of diversity—fractionalization and polarization—to matter for economic development over very long time frames. We not only assume that the growth-influencing traits of diversity become embedded in the local mentality, traditions and customs—in short, in local institutions—but also that big diversity shocks in a given period of time can become etched in the core characteristics of a territory and thus persist over very long time periods.

The implications of this assumption are twofold. First, US counties having received large inflows of both international and internal migrants stemming from a multitude of different origins more than a century ago should be significantly more prosperous today than those which displayed a more homogeneous population composition at the time. Second, we expect US counties marked by a highly polarized population composition during the Age of Mass Migration to have faced considerable barriers to the development of economic activity, deeply limiting their growth potential. Consequently, we assume historical fractionalization to be positively connected to current income levels across US counties, while historical degrees of polarization are likely to be negatively and significantly associated with them.

Moreover, in line with Ager and Brückner (2013b), we hypothesize that time will not significantly alter the impact of diversity on economic development. We assume a highly fragmented (highly polarized) society to retain its positive (negative) impact consistently over the short, the medium and the long term. Despite the fact that international migrants become American and internal migrants adopt local traits over time, their cultural baggage brought from their place of origin remains with them and is passed not just to the following generations, but especially to their chosen place of residence. As diversity becomes embedded in the core character of the county, it permanently influences its subsequent economic development path for decades.

### Model 1—Population Heterogeneity: The Case for Diversity

Model 1 is concerned with diversity measured as fractionalization and polarization. The model adopts the following form:

$$y_{i,t} = \alpha + \beta {\text{Fract}}_{{i,t_{0} }} + \lambda {\text{Pol}}_{{i,t_{0} }} + \partial X_{i,t - k} + \theta Z_{{i,t_{0} }} + \mu {\text{state}} + \varepsilon_{\text{is}}$$

where *y* represents the income per capita of county *i* in period *t* (*t *= 2010, 2000, …, 1900); *Fract* is the level of fractionalization in a given county *i* in *t*_0_, which corresponds to either 1880, 1900 or 1910; *Pol* is the degree of polarization in a given county *i* in *t*_0_; *X* is a vector of variables which are assumed to influence the level of development of any given county at time *t *− *k* (*k *= 10); *Z* represents a similar vector of factors which may have influenced the development of the county at time *t*_0_.^6^ Lastly, state depicts state controls taking into account unobservable state-specific effects and *ε* represents the error term clustered at the state level *s* to ensure robustness to arbitrary spatial correlation within one state. Our main coefficients of interest are $\beta$ and $\lambda$ describing the relationship of the two dimensions of diversity with economic development.

### Model 2—Population Homogeneity: The Case for Concentration

In order to assess the robustness of our results, Model 2 resorts to an index of concentration to reflect population homogeneity as the main independent variable of interest. All other variables remain the same as in Model 1. In this alternative set-up, the model adopts the following form:

$$y_{i,t} = \chi + \delta {\text{Conc}}_{{i,t_{0} }} + \phi X_{i,t - k} + \eta Z_{{i,t_{0} }} + \vartheta {\text{state}} + \omega_{\text{is}}$$

where *Conc* is defined as the level of concentration within the population of any given county *i* in *t*_0_ corresponding to either 1880, 1900 or 1910 and *ω* represents the error term clustered at the state level *s*. All other input variables refer to those presented in Model 1.

### Variables of Interest: Two Measures of Diversity and an Index of Concentration

#### Diversity

Following the two opposing strands of literature dealing with the link between diversity and economic growth, we resort to the two most commonly employed diversity indices to proxy population heterogeneity: fractionalization and polarization.

Fractionalization (i.e. Easterly and Levine 1997; Alesina et al. 2003) emphasizes the number of different groups within a population. It goes back to the work by the Soviet researchers Bruk and Apenchenko (1964) who crafted an index of ethnolinguistic fractionalization in the *Atlas Narodov Mira* (Atlas of the peoples of the world) based on the shares in total population of ethnolinguistic groups. The modified version of this index by Alesina et al. (2003) is used in this paper as our first indicator of diversity:

$${\text{Fract}}_{{i, t_{0} }} = 1 - \mathop \sum \limits_{g = 1}^{n} s_{{g,i, t_{0} }}^{2}$$

where ${\text{Fract}}_{{i, t_{0} }}$ is the degree of fractionalization in county $i$ at time $t_{0}$ where $s$ depicts the share of total population of origin group $g$ in county $i$ at time $t_{0}$. This index “captures the probability that two randomly selected individuals belong to different groups” (Campos and Kuzeyev 2007: 622). Hence, ${\text{Fract}}_{{i,t_{0} }}$ increases with the number of groups, taking on values between 0 and 1, with $1 - \sigma$ reflecting a highly fractionalized and $0 + \sigma$ a strongly homogeneous society, with $\sigma \to 0$.^7^ If each person in a territory belongs to a different group, the index reaches its theoretical maximum.

Polarization aims to capture the social tension and conflict dimension linked to a heterogeneous population. Esteban and Ray (1994, 1999), from a theoretical, and Montalvo and Reynal-Querol (2005a), from an empirical standpoint, argue that a highly polarized environment maximizes the risk of conflict. The measure of polarization is based on the family of indices developed by Esteban and Ray (1994, 1999), considering not only the number of ethnic groups within a society, but also the distances separating them and their individual size. According to this index type, the degree of polarization within a population increases as the distance between groups rises, but also when the number of groups increase or when there is convergence in group size. As the calculation of distance between ethnic groups is highly controversial, we follow Reynal-Querol (2002) for our index, assuming the absolute distance between two groups to be equal and discrete.^8^ The polarization index in this case “measures the normalized distance of a particular distribution of ethnic […] groups from a bimodal distribution” (ibid, p. 301) and is maximized when two highly distinguishable groups of equal size coexist within the same population.

The polarization index takes on the following form:

$${\text{Pol}}_{{i,t_{0} }} = 1 - \sum\limits_{g = 1}^{n} {\left( {\frac{{0.5 - s_{{g,i,t_{0} }} }}{0.5}} \right)_{{}}^{2} * s_{{g,i,t_{0} }} }$$

where ${\text{Pol}}_{{i,t_{0} }}$ is the degree of polarization in county $i$ at time $t_{0}$, and $s$ depicts the share of total population of origin group $g$ in county $i$ at time $t_{0}$. Within this particular specification, it is the size of the groups relative to each other that is of particular importance.

Both indices used in the analysis are aligned to the specifications of our dataset. Instead of ethnic groups as generally used in the literature, we consider the birthplaces of individuals—as defined by the US Census—living in a given county at $t_{0}$ as an indicator for different cultural groups. Birthplaces include both European countries and American states in order to properly account for international as well as for the high degree of internal migration prevailing at the time. As the historical US Census did not record the county of birth, but solely the state, our indicator does not pick up the bulk of the short-distance, intra-state internal migration. Only population groups of internal migrants which travelled large distances leaving their home state are included into the calculation of the index. We therefore take into consideration only the fraction of internal migrants which were identified to be significantly different to the local population (i.e. Merk 1978).^9^

**Fig. 4:**
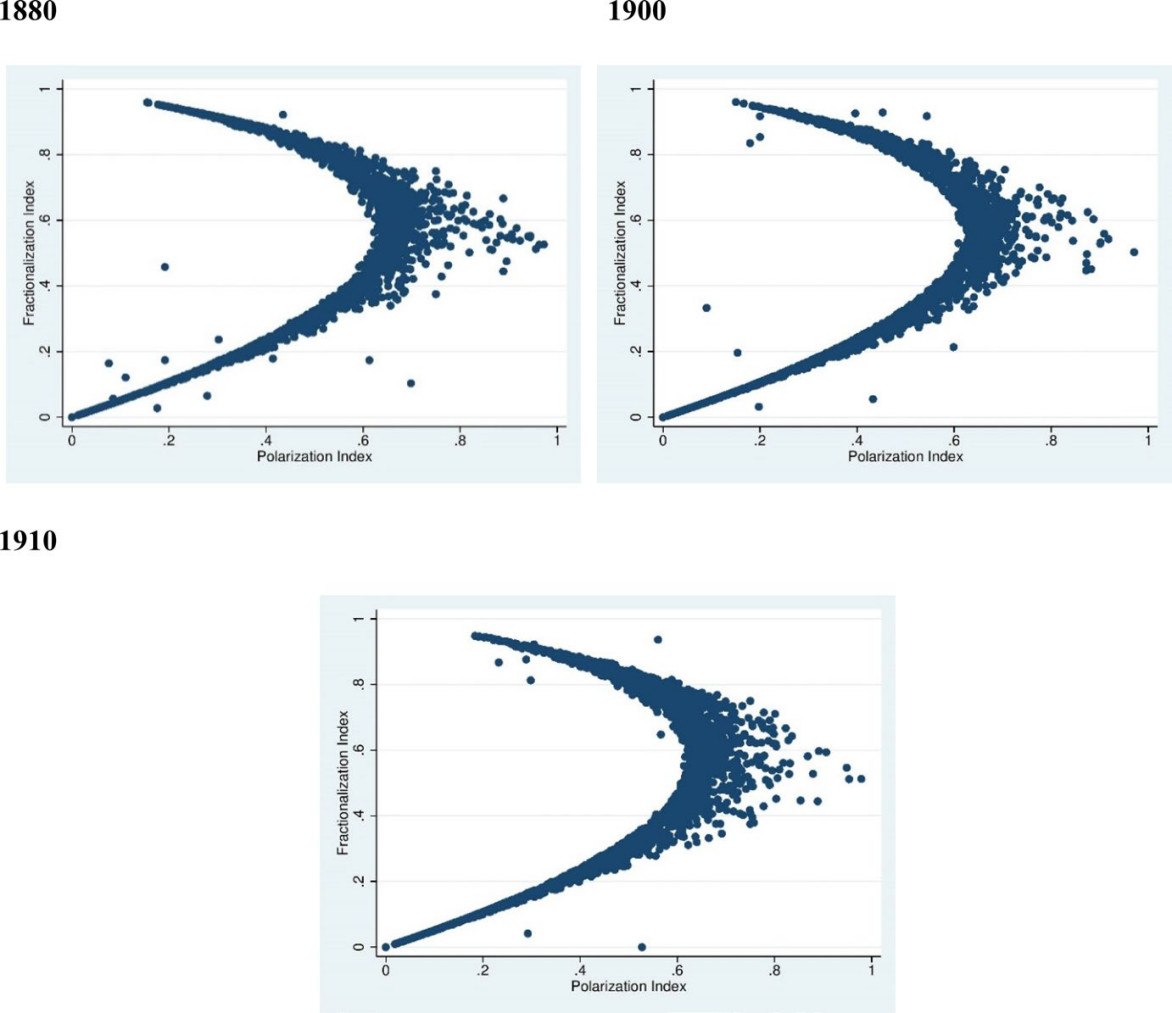
Fractionalization versus polarization for all three base years. *Source*: Own elaboration, using Ruggles et al. (2015) data

Figure 4 plots the relationship between polarization and fractionalization in US counties for all three base years: 1880, 1900 and 1910. It is important to note that varying the size of both indices does not reveal a consistent interdependency. Conditional on the degree of polarization, the extent of the correlation with fractionalization varies. Both indices are highly positively correlated at low levels of societal diversity, indicating that adding a further cultural group to an otherwise perfectly homogenous population increases the risk of polarization and conflict. However, as cultural heterogeneity increases, the positive relationship wanes and becomes irrelevant at medium levels of both polarization and fractionalization. The more a population becomes fragmented, the lower the societal standing and influence of a single population group, which reduces the societal polarization within a given county. At higher levels of fractionalization, the relationship between both indices turns strongly negative. Once above a certain fractionalization threshold, the addition of further cultural groups to a population significantly decreases the risk of polarization. This relationship is consistent across all three base years and in line with previous findings by Montalvo and Reynal-Querol (2005a, b), Ager and Brückner (2013a) or Bove and Elia (2017), underlining the validity of the data.

It is important to stress once again that both indices measure entirely different dimensions of diversity. While one focuses on the number of cultural groups leading to innovation, the other stresses their relative size, and thus the potential for provoking social unrest and conflict. Based on these highly distinct theoretical concepts, both indices thus identify independent and distinguishable effects of diversity on economic growth. From a theoretical standpoint, their joint inclusion in our empirical model minimizes the risk of omitted variable bias and allows us to capture a more accurate and encompassing effect of the multidimensional notion of diversity on economic growth. Concerns about the joint inclusion of both variables are addressed from an empirical standpoint in Fig. 4, which rules out the risk of biased results due to correlation issues. Following Ager and Brückner (2013a), Alesina et al. (2003), Montalvo and Reynal-Querol (2005a) and Gören (2014), we include both indices of fractionalization and polarization in our model, as both indices capture a different aspect of diversity.^10^

#### Concentration

The opposite of diversity is concentration, understood as the marked dominance of one group (based on place of origin) in a given territory. We employ this alternative variable of interest to assess the robustness of the results when analysing the diversity indices. The concentration index is defined as follows:

$${\text{Conc}}_{{i,t_{0} }} = \hbox{max} (s_{{g,i,t_{0} }} )$$

where ${\text{Conc}}_{{i,t_{0} }}$ is the degree of concentration within the population of county $i$ at time $t_{0}$, and $s$ depicts the share of total population of origin group $g$ in county $i$ at time $t_{0}$. According to this definition, the index always takes on the population share of the largest represented birthplace group within the population of the particular county $i$, thereby indicating the degree of concentration within a territory.

### Controls: Factors Influencing County Development at Two Different Points in Time

We introduce two sets of control variables into our model. The first group of control variables is vector $Z$ dating from the time of the big migration waves—1880, 1900 and 1910—and consists of factors which influenced a county’s attraction to immigrants at the time. The controls comprise mean income (as natural log), total population (as natural log), literacy rate, unemployment rate, female participation rate in the labour force, share of black population and the percentage of workers employed in agriculture. As these parameters are bound to have influenced the settlement decision of the individual migrants (see Jennissen 2003), we can assume they would also have exerted a strong impact on fractionalization and polarization at county level in the late nineteenth and early twentieth centuries. Furthermore, if certain population groups predominantly settled in wealthy regions and if this initial prosperity persisted over time, excluding the initial endowment factors of a county would lead to omitted variables and therefore biased estimates.

The second set of control variables in our model, vector $X$, represents the *t* − *k* time dimension, which corresponds to 10 years prior to the period considered in the dependent variable. Again, we control for factors influencing the economic development of the county, such as population size (as natural log), educational attainment, female participation in the labour force, unemployment, the share of black population, infant mortality and the share of the labour force employed in agriculture. We shift $X$ by 10 years in order to reduce the risk of reverse causality between diversity levels and income per capita.

### The Data

For the construction of the dependent variable, we employ income per capita data extracted from the US Bureau of Economic Analysis (BEA) database and the Current Population Survey tables (CPS) of the US Bureau of Labor Statistics (BLS), measured in US dollars. As income per capita was only available for the years 1950 onwards, we resorted to a proxy for the years 1900–1940 and used either the salary income (1940) or calculated an aggregated mean income at county level constructed using the median total income per occupation in 1950 dollars (1900–1930). The necessary input data for these proxies were extracted from the Integrated Public Use Microdata Series USA database (IPUMS) Version 6.0 (Ruggles et al. 2015). This database provides US microdata covering the censuses and American Community Surveys between 1790 and 2010.^11^ We use the natural log of income as dependent variable.

The main independent variables of interest, fractionalization and polarization as well as concentration were built using the birthplace data at county level of the years 1880, 1900 and 1910, extracted from the IPUMS USA database. The birthplaces of a weighted population sample of 5,791,531 individuals in 1880, 3,852,852 individuals in 1900 and 923,153 individuals in 1910 were aggregated and allocated to the counties of residence of the individual. As the number and size of US counties changed over the period of analysis (2875 counties in 1880, 3090 in 1900 and 3123 in 1910), we matched counties at the time of migration to their 2010 equivalent using US Census Bureau cartographic boundary files of the 48 continental states for each decade included in the analysis.

Data for the control variables were extracted from the IPUMS USA, the US BEA, the US BLS, the Inter-University Consortium for Political and Social Research (ICPSR) database as well as from the Centers for Disease Control and Prevention (CDC) databases. In cases involving microdata, the data for individuals were aggregated at the county level. With the exception of mean income and educational attainment, all variables followed the same calculation method based on the same available data points across all years in question. The variable expressing the aggregated mean income at county level in the late nineteenth and early twentieth century is constructed similarly to our dependent variable on the basis of the median total income per occupation (in 1950 dollars). Educational attainment is proxied by the percentage of people completing their college education for the years 1940–2000. From 1880 to 1930, we used the literacy rate per county as educational variable. A description of all variables is given in Appendix 1.

#### Instrumental Variable Estimation

Several endogeneity issues may arise when dealing with long-term migration data. While diversity may affect local GDP per capita, it is also likely that a higher GDP itself attracts more migrants, thereby increasing the level of diversity in the region. Consequently, the direction of causality remains ambiguous: GDP per capita on a regional level may be a function of diversity just as local diversity may be a consequence of local wealth. Moreover, when working with migration data, non-random spatial patterns in the distribution of migrants across space are likely to appear. Regional spillovers in migration may therefore generate clusters of counties with high levels of diversity. This spatial sorting would lead to endogeneity issues in our OLS regressions due to omitted variables. In order to address these endogeneity issues, we resort to instrumental variable (IV) estimation methods with the aim of revealing the true underlying effect of past diversity levels on income levels over time and to ensure the validity of the least squares estimations.^12^ We employ a shift-share methodology following Card (1999), Ottaviano and Peri (2006) and Saiz (2007). This instrument computes the estimated population composition of a county in 1880, 1900 and 1910 based on the population composition in a previous base year^13^ and the US growth rate per population group between base and target years. This implies using the share of inhabitants per birthplace within the population of a county in the base year and multiplying this share by the growth rate of that particular group within the US population for the time frame between base year and 1880, 1900 or 1910. Hence, we extrapolate predicted population shares under the assumption that migrants settle in areas where their predecessors had already established themselves. With these calculated predicted population shares, we then estimate an imputed fractionalization, polarization and concentration index for each county in the respective target year.

The use of the shift-share instrument is based on the assumption that highly diverse counties in the earlier years of the big migration waves developed a diversity buzz which became a pull factor for new migrants. With the use of the shift-share instrument, we assume these highly diverse counties to have remained attractive to incoming migrants in the following decades, implying also that any changes in the degree of diversity at county level would have been independent of county-specific shocks that may have taken place within the time frame in question.

The results of the Staiger and Stock (1997) test for weak instruments using the first-stage F-statistic of joint significance confirm that the shift-share variables for fractionalization, polarization and concentration are all strong instruments. The Kleibergen-Paap Wald F statistics in combination with the Stock and Yogo (2005) critical values for tests of weak instruments further support the validity of the instruments. The instruments reject the null of weak identification when testing at a nominal 5% significance level. Both the imputed polarization and fractionalization indices as well as the shift-share instrument for concentration are identified as strong across the three base years and the various time shifts of the dependent variable.

## Analysis

### The Long Term

The analysis starts with an evaluation of the long-term impact of diversity. Our first research question—whether population diversity levels generated in 1880, 1900 and 1910 are connected to county-level income per capita 100–130 years later—is first assessed by means of an ordinary least squares regression. As mentioned in the empirical approach, the model controls for wealth-influencing factors both at the time of migration and in recent years and includes state controls in order to control for state-specific unobserved factors influencing the counties’ prosperity (von Berlepsch et al. 2018). Table 2 reports the results of Model 1 for our two main variables of interest, fractionalization and polarization, for 1880, 1900 and 1910 with respect to income per capita levels in 2010.

**Table 2:** Long-term impact of diversity—OLS 1880, 1900 and 1910

Dep. variable: income p.c. 2010 (ln)	1880 OLS	1900 OLS	1910 OLS
Fractionalization^a^	0.144***	0.176***	0.155***
	(0.0501)	(0.0474)	(0.0323)
Polarization^a^	− 0.0365	− 0.0470	− 0.0301
	(0.0411)	(0.0376)	(0.0308)
Education 2000	0.0125***	0.0125***	0.0123***
	(0.000782)	(0.000826)	(0.000822)
Total population 2000 (ln)	0.00145	0.00296	− 0.00257
	(0.00566)	(0.00560)	(0.00562)
Share of black population 2000	− 0.00133***	− 0.000912*	− 0.00141***
	(0.000462)	(0.000489)	(0.000493)
Female participation 2000	− 0.000141	0.000245	0.000563
	(0.00117)	(0.00110)	(0.00107)
Unemployment 2000	− 0.0247***	− 0.0264***	− 0.0246***
	(0.00461)	(0.00421)	(0.00452)
Infant mortality 2000	− 8.41e−05	− 0.000161	− 0.000126
	(0.000322)	(0.000288)	(0.000286)
Agriculture 2000	− 0.000330	− 0.000405	− 0.000333
	(0.00208)	(0.00224)	(0.00226)
Mean income (ln)^a^	− 0.000603	− 0.000442	− 0.00535*
	(0.00333)	(0.00407)	(0.00285)
Literacy^a^	0.0976**	0.0368	0.0679
	(0.0395)	(0.0850)	(0.0499)
Total population (ln)^a^	− 0.0120**	− 0.0125	− 0.00806
	(0.00498)	(0.00761)	(0.00834)
Share of black population^a^	0.219***	0.173***	0.209***
	(0.0459)	(0.0375)	(0.0435)
Female participation^a^	0.0319	− 0.0287	0.000231
	(0.0895)	(0.0910)	(0.0513)
Unemployment^a^	− 0.00865	− 0.0468**	− 0.207
	(0.00959)	(0.0189)	(0.161)
Agriculture^a^	− 0.0687	0.000508	− 0.0963***
	(0.0531)	(0.0675)	(0.0204)
State controls	Yes	Yes	Yes
Observations	2825	3024	3094
*R*^2^	0.642	0.637	0.642

Robust standard errors in parentheses, clustered at state level

*** *p* < 0.01; ** *p* < 0.05; * *p* < 0.1

^a^Variables date from respective year of migration 1880, 1900 or 1910

The results in Table 2 unveil a positive long-term connection of country-level population diversity with current GDP per capita. The fractionalization index displays positive coefficients with significance levels below 1% across all three base years. The presence of large numbers of different groups according to place of origin in one county during the age of mass migration is strongly associated with higher levels of income in that county 100–130 years later. Polarization, by contrast, remains insignificant across all three base years. Hence, polarization at the height of the big migration waves seems detached from current levels of county wealth.

The signs and significance of the coefficients of the control variables reinforce the validity of the model, as they are in line with traditional studies on the determinants of growth. A good educational endowment in 2000 is connected to higher levels of income per capita in 2010. Conversely, levels of unemployment and the percentage of black population—a proxy for poverty—in 2000 are linked to lower county wealth.

Of the factors that may have affected decisions to migrate more than a century ago, few are still connected to county levels of development in recent years. The one exception is the share of black population in 1880, 1900 and 1910. In all cases, counties with a higher percentage of black people at the turn of the century have significantly higher levels of income per head today. The total population of a county in 1880, the level of unemployment in 1900, the proportion of agricultural employment in 1910 and the mean income in 1910 are negatively associated with income per head in 2010, while literacy in 1880 displays a positive and significant sign. Population diversity—measured as fractionalization—hence proves to have a considerably stronger association with future income levels than the large majority of other base year controls. Put differently, the results suggest that a highly diverse population is a better indicator of future regional wealth than, in particular, county wealth at the time of migration.

The results prove to be robust to the replacement of the diversity variables by a measure of group concentration, as indicated in Model 2 (Table 3). The concentration index is significant at the 1% level—as was the case of the fractionalization index in Table 2—although the association with income per capita in 2010 is, as expected, negative. Hence, US counties with a more homogeneous population composition (dominated by one large group, regardless of whether the group originates from abroad or from a different American state) more than a century ago seem to have endured a substantially worse economic trajectory over the last 100–130 years than those which attracted a large number of people stemming from different birthplaces. The largely homogenous population composition has hampered the emergence of innovation boosting conditions linked to the buzz of diversity.

**Table 3:** Long-term impact of concentration—OLS 1880, 1900 and 1910

Dep. variable: income p.c. 2010 (ln)	1880 OLS	1900 OLS	1910 OLS
Concentration ^a^	− 0.158***	− 0.175***	− 0.149***
	(0.0486)	(0.0467)	(0.0329)
Education 2000	0.0125***	0.0125***	0.0123***
	(0.000777)	(0.000831)	(0.000822)
Total population 2000 (ln)	0.00196	0.00284	− 0.00251
	(0.00572)	(0.00569)	(0.00576)
Share of black population 2000	− 0.00133***	− 0.000857*	− 0.00141***
	(0.000458)	(0.000492)	(0.000496)
Female participation 2000	− 0.000129	0.000223	0.000535
	(0.00115)	(0.00110)	(0.00108)
Unemployment 2000	− 0.0249***	− 0.0268***	− 0.0247***
	(0.00476)	(0.00428)	(0.00462)
Infant mortality 2000	− 4.60e−05	− 0.000160	− 0.000127
	(0.000320)	(0.000293)	(0.000290)
Agriculture 2000	− 0.000157	− 0.000259	− 0.000287
	(0.00207)	(0.00227)	(0.00223)
Mean income (ln)^a^	− 0.00108	− 0.000371	− 0.00504*
	(0.00342)	(0.00408)	(0.00289)
Literacy^a^	0.104**	0.0522	0.0754
	(0.0393)	(0.0926)	(0.0531)
Total population (ln)^a^	− 0.0116**	− 0.0111	− 0.00585
	(0.00470)	(0.00758)	(0.00816)
Share of black population^a^	0.224***	0.172***	0.208***
	(0.0441)	(0.0389)	(0.0427)
Female participation^a^	0.0339	− 0.0308	− 0.00295
	(0.0927)	(0.0888)	(0.0514)
Unemployment^a^	− 0.00965	− 0.0463**	− 0.207
	(0.00924)	(0.0193)	(0.167)
Agriculture^a^	− 0.0743	− 0.00275	− 0.0974***
	(0.0525)	(0.0614)	(0.0199)
State controls	Yes	Yes	Yes
Observations	2826	3024	3094
*R*^2^	0.643	0.636	0.641

Robust standard errors in parentheses, clustered at state level

*** *p* < 0.01; ** *p* < 0.05; * *p* < 0.1

^a^Variables date from respective year of migration 1880, 1900 or 1910

As far as both sets of control variables are concerned, there is nearly no change in either the significance levels or in the signs of the coefficients compared to those reported in Table 2.

In order to address potential endogeneity issues due to the risk of omitted variable bias as a result of spatial sorting, reverse causality or unaccounted economic shocks, an instrumental variable estimation is performed using the aforementioned shift-share methodology for all three main variables of interest: fractionalization, polarization (Table 4, columns 1, 2 and 3) and concentration (Table 4, columns 4, 5 and 6).

**Table 4:** Long-term impact of diversity and concentration—IV 1880, 1900 and 1910

Dep Var: Inc. p.c. 2010 (ln)	(1)	(2)	(3)	(4)	(5)	(6)
	1880 IV	1900 IV	1910 IV	1880 IV	1900 IV	1910 IV
Fractionalization^a^	0.371***	0.391***	0.271***			
	(0.0997)	(0.123)	(0.0810)			
Polarization^a^	− 0.175**	− 0.165*	− 0.100*			
	(0.0784)	(0.0941)	(0.0570)			
Concentration^a^				− 0.375***	− 0.389***	− 0.261***
				(0.102)	(0.115)	(0.0683)
Education 2000	0.0125***	0.0126***	0.0123***	0.0124***	0.0126***	0.0124***
	(0.000782)	(0.000857)	(0.000806)	(0.000767)	(0.000880)	(0.000811)
Total population 2000 (ln)	0.00265	0.00107	− 0.00327	0.00272	0.000932	− 0.00302
	(0.00518)	(0.00594)	(0.00510)	(0.00532)	(0.00623)	(0.00540)
Black population 2000	− 0.00160***	− 0.00116**	− 0.00145***	− 0.00156***	− 0.00106**	− 0.00146***
	(0.000451)	(0.000479)	(0.000456)	(0.000437)	(0.000502)	(0.000467)
Female participation 2000	− 4.24e−05	0.000852	0.000747	9.59e−05	0.000814	0.000752
	(0.00115)	(0.000995)	(0.000993)	(0.00111)	(0.00102)	(0.00100)
Unemployment 2000	− 0.0227***	− 0.0243***	− 0.0248***	− 0.0242***	− 0.0255***	− 0.0249***
	(0.00472)	(0.00390)	(0.00440)	(0.00480)	(0.00427)	(0.00461)
Infant mortality 2000	− 0.000113	− 3.90e−05	− 0.000212	− 1.49e−05	− 5.21e−05	− 0.000216
	(0.000336)	(0.000303)	(0.000287)	(0.000317)	(0.000308)	(0.000293)
Agriculture 2000	9.45e−05	− 0.00109	− 0.000798	0.000716	− 0.000661	− 0.000415
	(0.00220)	(0.00212)	(0.00228)	(0.00228)	(0.00235)	(0.00232)
Income (ln)^a^	− 0.00337	− 0.00617	− 0.00764**	− 0.00367	− 0.00634	− 0.00749**
	(0.00364)	(0.00431)	(0.00333)	(0.00382)	(0.00448)	(0.00337)
Literacy^a^	0.0451	0.0221	0.0302	0.0694	0.0476	0.0523
	(0.0537)	(0.0783)	(0.0499)	(0.0492)	(0.0939)	(0.0536)
Total population (ln)^a^	− 0.0171***	− 0.0106	− 0.0112	− 0.0150***	− 0.00818	− 0.00769
	(0.00522)	(0.00929)	(0.00880)	(0.00496)	(0.00908)	(0.00822)
Black population^a^	0.226***	0.196***	0.212***	0.254***	0.204***	0.218***
	(0.0411)	(0.0362)	(0.0403)	(0.0405)	(0.0380)	(0.0394)
Female participation^a^	0.0533	− 0.0315	0.0115	0.0278	− 0.0338	0.00928
	(0.0875)	(0.0929)	(0.0519)	(0.0924)	(0.0903)	(0.0513)
Unemployment^a^	− 0.00115	− 0.0486**	− 0.225	− 0.00603	− 0.0456**	− 0.218
	(0.0128)	(0.0214)	(0.150)	(0.0105)	(0.0221)	(0.158)
Agriculture^a^	− 0.0650	0.0238	− 0.0958***	− 0.0716	0.0344	− 0.0913***
	(0.0543)	(0.0763)	(0.0196)	(0.0545)	(0.0667)	(0.0189)
State controls	Yes	Yes	Yes	Yes	Yes	Yes
Observations	2817	2826	3067	2820	2827	3069
First-stage F-statistic	22.63	36.95	99.28	87.19	74.10	213.74

Robust standard errors in parentheses, clustered at state level

*** *p* < 0.01; ** *p* < 0.05; * *p* < 0.1

^a^Variables date from respective year of migration: 1880, 1900 or 1910

Table 4 reports a positive and strongly significant impact of high levels of fractionalization in all three base years on income per capita levels in 2010 supporting the validity of previous results. In contrast to the OLS regressions, the polarization index, while remaining negative, becomes significant at the 5% level for 1880 and the 10% for 1900 and 1910, respectively. This proves that once we control for endogeneity issues and correct potentially biased estimators, diversity reveals its true underlying two-dimensional long-term impact on income per capita levels. On the one hand, the presence of a large number of groups and, thus, considerable population diversity (high fractionalization) is an important factor behind the long-term economic dynamism of places in the US, provided the diverse groups are not too polarized and, therefore, able to communicate with one another (low polarization). By contrast, highly homogeneous societies have experienced much lower economic dynamism over the long term (Table 4, regressions 4, 5 and 6). The signs and level of significance of the control variables remain virtually unchanged from those reported in Tables 2 and 3.

### The Dynamic Impact of Diversity

The second part of the analysis is dedicated to examining the dynamic impact of diversity on income levels. Starting with income levels in 1900, the dependent variable in Model 1 is changed each time by 10 years in order to account for potential changes in the influence of original population diversity on income per head. For this part of the analysis, however, vector $Z$, including the base year controls, is dropped from the estimation in order to avoid issues of multicollinearity in the earlier years considered. By means of an ordinary least squares regression, followed again by two rounds of robustness checks, we seek to analyse whether the impact of diversity on county-level income per head varies over time. The results of the analysis are reported in Table 5.

**Table 5:** The dynamic effect of diversity and concentration (OLS) (Detailed estimation results including control variable coefficients can be made available upon request)

Var. of interest	Base year	Dep var.: income	(1)	(2)	(3)	(4)	(5)	(6)	(7)	(8)	(9)	(10)	(11)
			1900	1910	1920	1930	1940	1950	1960	1970	1980	1990	2000
Diversity	1880	Fractionalization	0.247***	0.199*	0.087	0.012	0.0042	0.161***	0.236***	0.233***	0.300***	0.282***	0.322***
			(0.057)	(0.114)	(0.073)	(0.064)	(0.069)	(0.0555)	(0.0477)	(0.0510)	(0.0668)	(0.0605)	(0.0691)
		Polarization	− 0.078	− 0.028	− 0.054	− 0.046	− 0.059	− 0.0269	− 0.0864**	− 0.106*	− 0.164**	− 0.123*	− 0.132*
			(0.054)	(0.073)	(0.042)	(0.037)	(0.048)	(0.0430)	(0.0393)	(0.0541)	(0.0614)	(0.0673)	(0.0712)
		Observations	2835	2848	2858	2844	2561	2817	2870	2857	2865	2865	2871
		*R*^2^	0.571	0.551	0.587	0.624	0.626	0.844	0.802	0.634	0.490	0.432	0.303
	1900	Fractionalization		0.474***	− 0.036	− 0.0155	− 0.068	0.185**	0.273***	0.289***	0.244**	0.254**	0.274***
				(0.081)	(0.069)	(0.071)	(0.076)	(0.0802)	(0.0864)	(0.0969)	(0.102)	(0.116)	(0.101)
		Polarization		− 0.126*	− 0.015	− 0.058	− 0.048	0.0161	− 0.0243	− 0.0855	− 0.102	− 0.0704	− 0.0704
				(0.069)	(0.052)	(0.047)	(0.057)	(0.0537)	(0.0533)	(0.0667)	(0.0847)	(0.0933)	(0.0879)
		Observations		3046	3094	3070	2750	3046	3103	3085	3098	3098	3103
		*R*^2^		0.542	0.589	0.628	0.627	0.835	0.794	0.606	0.476	0.405	0.300
	1910	Fractionalization			− 0.049	0.0053	− 0.016	0.160**	0.159***	0.157**	0.198***	0.215***	0.283***
					(0.058)	(0.055)	(0.070)	(0.0630)	(0.0580)	(0.0724)	(0.0641)	(0.0721)	(0.0602)
		Polarization			− 0.019	− 0.054	− 0.095*	0.0162	0.0133	− 0.000155	− 0.0365	− 0.0354	− 0.0569
					(0.048)	(0.044)	(0.052)	(0.0633)	(0.0574)	(0.0739)	(0.0617)	(0.0671)	(0.0555)
		Observations			3117	3089	2757	3071	3128	3111	3123	3123	3128
		*R*^2^			0.591	0.628	0.628	0.833	0.788	0.600	0.474	0.400	0. 305
Concentration	1880	Concentration	− 0.250***	− 0.209**	− 0.054	0.022	0.025	− 0.158***	− 0.204***	− 0.199***	− 0.219***	− 0.205***	− 0.218***
			(0.069)	(0.102)	(0.068)	(0.059)	(0.062)	(0.0578)	(0.0476)	(0.0471)	(0.0605)	(0.0601)	(0.0647)
		Observations	2836	2849	2859	2845	2561	2818	2872	2859	2867	2867	2873
		*R*^2^	0.565	0.551	0.587	0.624	0.625	0.844	0.800	0.631	0.482	0.423	0.292
	1900	Concentration		− 0.471***	0.075	0.065	0.135	− 0.236***	− 0.319***	− 0.306***	− 0.183**	− 0.200**	− 0.195**
				(0.067)	(0.075)	(0.079)	(0.081)	(0.0829)	(0.0730)	(0.0872)	(0.0786)	(0.0831)	(0.0779)
		Observations		3046	3094	3070	2750	3046	3104	3086	3099	3099	3104
		*R*^2^		0.541	0.590	0.628	0.627	0.835	0.793	0.606	0.471	0.398	0.291
	1910	Concentration			0.086	0.035	0.098	− 0.209***	− 0.203***	− 0.184***	− 0.180***	− 0.196***	− 0.240***
					(0.055)	(0.054)	(0.069)	(0.0500)	(0.0420)	(0.0486)	(0.0564)	(0.0513)	(0.0545)
		Observations			3117	3090	2757	3072	3129	3112	3124	3124	3129
		*R*^2^			0.591	0.628	0.626	0.833	0.788	0.599	0.471	0.396	0.296
		Lag. contr.	Yes	Yes	Yes	Yes	Yes	Yes	Yes	Yes	Yes	Yes	Yes
		Base year contr.	No	No	No	No	No	No	No	No	No	No	No
		State controls	Yes	Yes	Yes	Yes	Yes	Yes	Yes	Yes	Yes	Yes	Yes

Robust standard errors in parentheses, clustered at state level

*** *p* < 0.01; ** *p* < 0.05; * *p* < 0.1

The results point towards an enduring and positive association between population diversity and local income levels in the US. With the exception of the 1920s to 1940s, heavily affected by the great depression and both world wars, the link between fractionalization and income per capita at the county level remains positive and strong, with no evidence of a waning or shifting connection over time. As in Table 2, with the exception of 1 year in the 1900 base year regression, there is no significant connection between population polarization and income levels.

To test the validity of these results, we conduct the same exercise substituting fractionalization and polarization by concentration levels within the county population (Table 5). In line with the previous long-term results (Table 3), a strong negative association between high levels of concentration and regional income levels emerges not only in the long term, but also in the short and medium term. Similarly, the effect of concentration is negative and significant over the whole 100-year time frame considered, with the exception of 1920–1940.

Both OLS regressions emphasize the importance of a county’s population composition at the time of the great migration waves to the US for its subsequent economic development. The results suggest that counties which failed to attract a large variety of groups from different origins—both international and national—seem to have suffered negative economic consequences for more than a century, as indicated by the significantly lower income levels, than those counties that succeeded in establishing vibrant and diverse communities.

Is this dynamic connection purely an association or is there a causal relationship? To answer this question, we resort to the use of an instrumental variable estimation, using, once again, a shift-share instrument. The results for both Models 1 and 2 are displayed in Table 6.

**Table 6:** The dynamic effect of diversity and concentration (IV) (Detailed estimation results including control variable coefficients can be made available upon request)

Var. of interest	Base year	Dep var.: income	(1)	(2)	(3)	(4)	(5)	(6)	(7)	(8)	(9)	(10)	(11)
			1900	1910	1920	1930	1940	1950	1960	1970	1980	1990	2000
Diversity	1880	Fractionalization	1.961***	0.899***	− 0.081	− 0.050	− 0.118	0.227***	0.425***	0.450***	0.396***	0.414***	0.553***
			(0.181)	(0114)	(0.1005)	(0.095)	(0.110)	(0.0708)	(0.0663)	(0.0727)	(0.0798)	(0.0848)	(0.0914)
		Polarization	− 0.893***	− 0.407***	− 0.023	− 0.073	− 0.053	0.0593	− 0.0849	− 0.188***	− 0.221***	− 0.214**	− 0.296***
			(0.172)	(0.110)	(0.092)	(0.086)	(0.104)	(0.0679)	(0.0665)	(0.0729)	(0.0805)	(0.0835)	(0.0858)
		Observations	2831	2844	2849	2832	2553	2806	2857	2845	2852	2852	2858
		First-stage F-stat	73.55	78.41	70.38	87.16	61.99	72.93	82.92	82.88	76.85	77.17	74.78
	1900	Fractionalization		0.661***	0.129	0.0332	0.054	0.327***	0.417***	0.451***	0.553***	0.463***	0.539***
				(0.119)	(0.107)	(0. 099)	(0.101)	(0.0792)	(0.0683)	(0.0745)	(0.0829)	(0.0825)	(0.0916)
		Polarization		− 0.332***	− 0.062	− 0.065	− 0.104	− 0.105	− 0.106*	− 0.191***	− 0.297***	− 0.198***	− 0.236***
				(0.095)	(0.087)	(0.0802)	(0.085)	(0.0674)	(0.0579)	(0.0636)	(0.0708)	(0.0726)	(0.0791)
		Observations		2848	2853	2836	2557	2810	2861	2849	2856	2856	2862
		First-stage F-stat		135.81	136.13	145.29	123.39	153.70	151.99	156.91	142.44	144.29	137.57
	1910	Fractionalization			− 0.060	− 0.018	− 0.075	0.220***	0.378***	0.409***	0.355***	0.349***	0.358***
					(0.072)	(0.067)	(0. 072)	(0.0499)	(0.0511)	(0.0570)	(0.0594)	(0.0611)	(0.0607)
		Polarization			− 0.019	− 0.064	− 0.058	0.0194	− 0.0675	− 0.151***	− 0.185***	− 0.150***	− 0.140**
					(0. 061)	(0.057)	(0.060)	(0.0454)	(0.0446)	(0.0502)	(0.0553)	(0.0559)	(0.0555)
		Observations			3090	3064	2744	3041	3096	3079	3091	3091	3096
		First-stage F-stat			246.59	261.98	255.31	282.16	279.32	279.97	286.17	293.68	309.42
Concentration	1880	Concentration	− 1.895***	− 0.868***	0.223	0.188	0.259*	− 0.330**	− 0.456***	− 0.425***	− 0.245*	− 0.249*	− 0.318**
			(0.272)	(0.154)	(0.148)	(0.150)	(0.147)	(0.145)	(0.120)	(0.129)	(0.141)	(0.136)	(0.154)
		Observations	2836	2849	2854	2837	2557	2811	2862	2850	2857	2857	2863
		First-stage F-stat	85.22	80.40	81.84	94.15	95.99	93.91	101.21	111.46	106.47	104.99	98.26
	1900	Concentration		− 0.618***	− 0.066	0.041	0.042	− 0.356***	− 0.429***	− 0.408***	− 0.407***	− 0.355***	− 0.398***
				(0.194)	(0.144)	(0.124)	(0.125)	(0.131)	(0.0974)	(0.106)	(0.125)	(0.109)	(0.133)
		Observations		2849	2854	2837	2557	2811	2862	2850	2857	2857	2863
		First-stage F-stat		63.38	65.03	72.04	70.39	76.30	73.25	79.03	70.62	69.16	65.78
	1910	Concentration			0.157	0.123	0.190*	− 0.297***	− 0.410***	− 0.380***	− 0.227**	− 0.235**	− 0.218**
					(0.098)	(0.100)	(0.1003)	(0.114)	(0.0992)	(0.110)	(0.105)	(0.109)	(0.102)
		Observations			3094	3069	2748	3046	3101	3084	3096	3096	3101
		First-stage F-stat			238.21	307.38	235.88	298.02	254.42	281.25	300.42	293.77	293.81
		Lag. contr.	Yes	Yes	Yes	Yes	Yes	Yes	Yes	Yes	Yes	Yes	Yes
		Base year contr.	No	No	No	No	No	No	No	No	No	No	No
		State controls	Yes	Yes	Yes	Yes	Yes	Yes	Yes	Yes	Yes	Yes	Yes

Robust standard errors in parentheses, clustered at state level

*** *p* < 0.01; ** *p* < 0.05; * *p* < 0.1

Again, and with the exception of the period between 1920 and 1940, the results depict a strong and robust association across time between population diversity and regional income levels. As in the OLS estimations, the coefficient for the fractionalization index remains, across all three base years, positive and highly significant at the 1% level. In contrast to the OLS regressions, the use of an IV estimation makes the coefficient of polarization significant for the early (1900–1910) and later years (1960–2000) of the analysis. Polarization has, as expected, a negative influence on county-level economic development in line with Alesina et al. (2003), Montalvo and Reynal-Querol (2005a,b), or Ager and Brückner (2013a). Wherever strong barriers across place-of-birth origins were evident among population groups at the time of the great migration, local development has lagged behind.

**Fig. 5:**
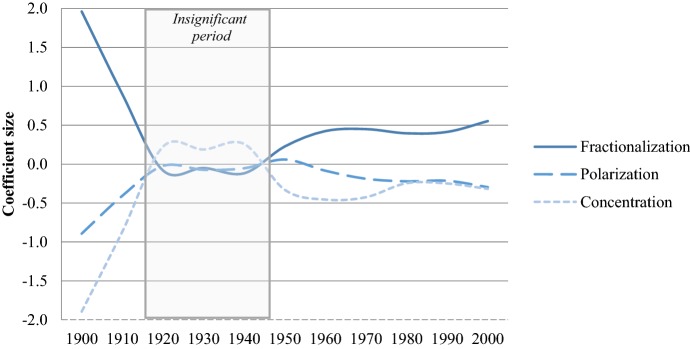
Evolution of coefficients for population fractionalization, polarization and concentration over time (IV, base year 1880). *Source*: Own elaboration

Moreover, we now find a dynamic effect related to the size of the coefficients. This is particularly evident in the case of the 1880 and 1900 base year regressions. Columns 1 and 2 (income in 1900 and 1910 as dependent variables) in Table 6 display coefficients up to 6 times larger than those presented in columns 10 or 11 (1990 and 2000). The results indicate that high levels of fractionalization and polarization in the composition of a population had a more powerful effect on income levels within the first 10–30 years, while, in the longer term, despite remaining significant, the extent of this effect becomes significantly smaller (Fig. 5). Hence, a high degree of population diversity, generated by mass internal and international migration at the turn of the twentieth century, is at the origin of some sort of diversity *buzz*. Such local *buzz* produced fertile grounds for long-term increases in productivity and innovation (Jacobs 1961, 1969). But the impact of population diversity has not been constant over time. The influence of diversity on county-level wealth was particularly strong during the years when migrants were still economically active and kept the local population culturally diverse. As long as the different population groups remained clearly distinct from one another and immersed in the culture of their home countries and home regions, the economic impact of diversity remained high. The assimilation of migrants and, especially, of their children into the American melting pot reduced population diversity and, consequently, attenuated its positive economic effects. As the cultural distance between previously highly different population groups decreased with adaptation to the American way of life, the economic premium linked to past local diversity waned. However, the positive effect of past diversity *buzz* did not disappear completely: formerly diverse counties remained more dynamic over time than counties that had, by and large, stayed mostly homogeneous in their population composition. Hence, diversity shocks at local level seem to have triggered economic mechanisms that became engraved in the territory and have proven enduring—leaving traces that can still be detected more than a century after the initial shock took place. In spite of the assimilation of former migrants into American culture, the rapid “Americanization” of their children, and the loss of local diversity over successive generations, high historical population diversity levels in the late nineteenth and early twentieth century still affect current local economic development across the US. Diversity linked to migration has left a very long-lasting trace on local wealth, which is still measurable in terms of higher average income levels today.

Replacing fractionalization and polarization with concentration yields results which are almost the reverse carbon copy of the fractionalization coefficients. Concentration proves yet again to have a negative, enduring, and strongly robust impact on local economic development. Just like the indexes of fractionalization and polarization, high levels of concentration within a county’s population reveal a dynamic impact on income levels over time. The large coefficients in the short term decrease over time, despite keeping strong significance levels throughout.

The above findings reinforce and extend the findings of Hong and Page (2001), Florida (2002), and Niebuhr (2010) that more diverse places, measured by the number of population groups, are more innovative and productive than more homogeneous places. Diversity fosters economic growth—not only in the short term, but also in the medium term and even in the very long term. However, channels for dialogue between the different groups need to be established, as the relative size and distance between groups of different origins interacting in a territory prove to be detrimental for sustainable economic development. If the lines separating groups are too deep and insuperable, communication lines fail, bridging between groups becomes difficult, resulting in social unrest and conflict, highly polarized societies, and thus low economic growth.

## Conclusion

The question of whether and how population diversity impacts the economic trajectory of territories has recently attracted increasing attention (i.e. Easterly and Levine 1997; Alesina and La Ferrara 2005; Ottaviano and Peri 2006; Gören 2014; Alesina et al. 2016; Bove and Elia 2017). The literature dealing with the topic has focused on a multitude of factors, ranging from the labour force set-up and skill endowments to the provision of public goods. Two opposing views have emerged—one referring to diversity as growth-enhancing, the other as growth-reducing. Each view is to some extent dependent on the respective diversity indicator employed. The most frequently used are population fractionalization and measures of polarization and segregation.

Most analyses have, however, typically considered the short-term economic impact of diversity rather than evaluating its effects over longer time frames. Despite an undeniable effect on growth over the short term, whether past population diversity levels still affect economic outcomes over the medium or long term and whether there is a time-varying impact on regional economic prosperity remains an almost untouched area within the scientific literature. This paper has aimed to fill in the gap. The objective has been to assess the extent to which diversity, measured as two-dimensional notion of fractionalization and polarization, in the population composition of US counties during the Age of Mass Migration between 1880 and 1910 has left an imprint on the region’s economic development and whether that potential imprint can still be felt today, more than a century later. It also evaluates whether the dimension and the direction of the impact of diversity on economic development trends over time, by considering the impact of diversity over a period of 130 years, shifting the focus of the analysis 10 years at a time between 1900 and 2000.

The results of the analysis identify the presence of a strong and very long-lasting impact of diversity on county-level economic development. Counties that attracted migrants from very diverse national and international origins over a century ago are significantly richer today than those that were marked by a more homogeneous population at the time. Highly diverse counties after the big migration waves of the late nineteenth and early twentieth centuries strongly benefited from the enlarged skill set, the different perspectives and experiences the arriving migrants brought with them and from the interaction among those different groups. The result was the surge of new ideas and of a newfound dynamism which quickly became translated into lofty short-term economic gains. These gains proved durable and, albeit in a reduced way, can still be felt today.

Yet, the benefits of diversity came with a strong caveat: the gains of having a large number of groups from different origins within a territory (fractionalization) only materialize if the diverse groups are able to communicate with one another (low polarization). Deep cut lines separating the groups (high polarization) emerge as an important barrier for economic development. Hence, diversity becomes a double-edged sword: it works only if the different groups can interact, that is, if the “melting pot” really happens. Where it is not possible to build a dialogue between the different groups, where bridging does not occur, groups and communities remain in their own physical or mental ghettoes, undermining any economic benefits from a diverse environment.

In the US context, the benefits from diversity have remained over time. Where high levels of diversity were coupled with “bridging” across groups—high population fractionalization with low polarization—economic gains arose that were felt in the short, medium and long term. With the exception of the highly turbulent 1920s to 1940s, a strongly positive and robust association between fractionalization and regional income levels, as well as a negative association of polarization, is in evidence in the analysis. The only change in this enduring relationship is that both connections, while remaining strongly statistically significant, become weaker after the 1920s. While the initial spark of diversity at the turn of the twentieth century was a strong booster of economic dynamism for a period of between 10 and 30 years, its impact, albeit decreasing, has not yet faded entirely. A strong diversity residue remains.

We can only speculate about the reasons of why this is the case. As successive generations of migrants have blended into the American “melting pot” and often moved away from where their ancestors settled, the seeds of diversity may have grown roots not only in local institutions, but also in places. Diversity, in those places where it facilitated the bridging among groups more than a century ago, has in all likelihood generated more welcoming, vibrant, entrepreneurial and innovative territories. This vibrancy has, in a way, become embedded in the very core of the territory, a factor which guarantees that transformations which took place a very long time ago are still felt today. However, further case-study based, anthropological research will be needed in order to firmly prove this point.

The results of the analysis also have implications for policy. Even though the conditions and circumstances today do not correspond to those in the USA in the late nineteenth and early twentieth centuries, our results appeal for pause and thought in a period when migration policies are fast changing and have often become driven by anti-system and populist parties and the tabloid press. At a time when many developed countries are rapidly closing down their borders to immigration, trying to shield what—particularly in the case of Europe and Japan—are still rather homogeneous populations from external influences and the perceived security, economic and welfare threats often unjustly associated with migrants, restricting migration will limit diversity and is bound to have important and long-lasting economic consequences. By foregoing new migration, wealthy societies may be jeopardizing, as our research shows, not only the short-term positive impact associated with greater diversity, but also the enduring positive influence of diversity on economic development. The large, positive and persistent impact of societal diversity on economic development seen in the United States would therefore be difficult to replicate—something that ageing and lethargic societies across the developed world cannot relinquish. However, if migration is to be encouraged, it is of utmost importance that mechanisms facilitating the dialogue across groups and hence the integration of migrants are in place to guarantee that diversity is transformed into higher and durable economic activity over the short, medium and long term.

### Electronic supplementary material

Below is the link to the electronic supplementary material.

Supplementary material 1 (PDF 296 kb)

## Appendix 1: Variable descriptions

**Table:**

Variable	Description	Source
Income (ln)	Natural log of income levels of county *i* in year *t*	
	1950–2010: income per capita in current dollars—not adjusted for inflation	US BEA, US BLS
	1940: family wage and salary income in current dollars—not adjusted for inflation	IPUMS USA
	1900–1930: aggregated mean income constructed on the basis of median total income per occupation in hundreds of 1950 dollars	IPUMS USA
Fractionalization	Level of fractionalization in county *i* in year *t*_0_	IPUMS USA
		Own construction
Polarization	Level of polarization in county *i* in year *t*_0_	IPUMS USA
		Own construction
Concentration	Level of concentration in county *i* in year *t*_0_	IPUMS USA
		Own construction
Shiftshare_diversity^a^	Fractionalization index-based shiftshare in year *t*_0_	IPUMS USA
		Own construction
Shiftshare_polarization^a^	Polarization index-based shiftshare in year *t*_0_	IPUMS USA
		Own construction
Shiftshare_concentration^a^	Concentration index-based shiftshare in year *t*_0_	IPUMS USA
		Own construction
Education	1940–2000: percentage of population of county *i* with college degree	ICPSR
Literacy	1880–1930: literacy rate in county *i*	IPUMS USA
Total population (ln)	Natural log of total population in county *i*	ICPSR
Share of black population	Percentage of black population of county *i*	ICPSR and IPUMS USA
Female participation	Female participation rate in the labour force in county *i*	ICPSR and IPUMS USA
Unemployment	Unemployment rate in county *i*	ICPSR, US BEA and US BLS
Infant mortality	Infant mortality rate in county *i*	CDC and ICPSR
Agriculture	Percentage of the labour force employed in agriculture in county *i*	ICPSR, US BEA and US BLS
Mean income (ln)	Natural log of mean income of population in county *i* in 1880, 1900 and 1910	ICPSR and IPUMS USA
State controls	State dummies	Own construction

^a^Base years: 1870 for 1880 and 1880 for subsequent years

## Footnotes

1 A thorough discussion of the unique contribution of the manuscript to the existing literature is presented in Section 1 of Supplementary Online Material.

2 In peak years, the annual inflow rate of immigrants reached heights of around 1.5% of the total population at the time (Kim 2007).

3 With the notable exception of the Chinese after the Chinese Exclusion Act of 1882 (Frazier and Margai 2010).

4 The situation in 1880 and 1900 was roughly similar. Maps are available upon request.

5 The calculation of the fractionalization index will be explained in detail in Sect. 4.

6 In order to avoid multicollinearity problems, this vector is only included when analysing the long term. For the short-term regression analysis, this vector is excluded.

7 $\sigma$ represents any small positive number so that ${\text{Fract}}_{{i, t_{0} }}$ becomes arbitrarily close to 1 or 0, respectively.

8 One of the few papers estimating the distance between ethnic groups is Fearon (2003). Language is used as a proxy of cultural distance.

9 The reasons for the inclusion in the diversity index of both long-distance internal and external migrants are developed in greater detail in Section 2 of Supplementary Online Material.

10 A more detailed discussion of the reasons for the introduction of the fractionalization and polarization indices together in the same regression is presented in Section 3 of Supplementary Online Material.

11 The American Community Survey was only initiated in 2005.

12 An extensive discussion of the spatial nature of the data and of the potential existence of spatial dependency is developed in Section 4 of Supplementary Online Material.

13 Diversity levels in 1880 are instrumented by the population composition in 1870. For 1900 and 1910 values, we used 1880 base values due to the significantly larger data sample available.
