# Supplementary material for: Does Population Diversity Matter for Economic Development in the Very Long Term? Historic Migration, Diversity and County Wealth in the US
Source: Eur J Popul. 2018 Dec 4;35(5):873–911. doi: 10.1007/s10680-018-9507-z (PMC6882754; doi:10.1007/s10680-018-9507-z)
Supplement: Supplementary file 1 — Supplementary material 1 (PDF 296 kb) [file 10680_2018_9507_MOESM1_ESM.pdf]

# Does population diversity matter for economic development in the very long-term?

# Historic migration, diversity and county wealth in the US

## *Supplementary Online Material*

### Section 1

***The contribution of the manuscript to the existing literature***

What are the key contributions of the article to existing knowledge? How does this paper link to the new, but emerging literature on the long-term economic impact of migration?

Research on the very long-term impact of migration at county level was pioneered by Rodríguez-Pose and von Berlepsch's (2014) in an article published in the *Annals of the Association of American* Geographers. This paper deals with the long-term impact of overall migration on economic outcomes more than 100 years later. The authors examine the extent to which the settlement pattern of migrants to the United States at the end of the 19th century/beginning of the 20th century left a legacy on the economic development of US counties and whether this legacy still endured more than a century later. The model looked at the share of migrants (regardless of nationality) per county in 1880, 1900, 1910 and its association with recent levels of local development (proxied by per-capita income in 2005). Diversity was not covered in any sort of way, in an article that focused on the overall size of the migrant population of a county as a share of the total population – independent of the composition of migrants, their birthplaces or ethnicities. The emphasis was simply placed on the presence and share of foreign immigrants.

This research was followed by the same authors in an article published in *Economic Geography* (Rodríguez-Pose and von Berlepsch, 2015). The authors examine whether the national origin of migrants made a difference for long-term economic development. In this piece of research the settlement patterns of international migrants according to their specific national origin were explored, with the aim of assessing whether a large share of i.e. Irish, German or Italian settlers had left a distinctive impact on long term economic development and whether this impact changed according to national origin. In other words, the authors used a dataset on birthplaces of migrants from 1880 and 1910 and analysed if the nationality of the migrants settling in a given US county more than a century ago was decisive for the subsequent growth path of the territory. The focus of this paper was thus the size of the migrant community from one particular country within a county, measured as the share of

total county population, not the size of the entire migrant community nor its composition. Diversity aspects within the migrant population were not treated in any way.

With respect to other relevant literature in the field of diversity and its dynamic impact on economic development over time, the present article has four unique points which clearly distinguish it from previous studies. Firstly, the article deals with the long-term impact of migrant diversity on a decade by decade basis for a period of 130 years (1880-2010). Secondly, it defines diversity as a two-dimensional notion, incorporating both aspects of diversity – cultural fractionalization and cultural polarization – which have been discussed in the theoretical as well as the short-term empirical literature and are shown to have a distinguishable impact on economic growth, but have never been covered over the very long run. Thirdly, both internal as well as external migration are taken into account for the measure of diversity. Lastly, rather than evaluating a cross-country sample, the paper covers to graphical units as a great deal of granularity, incorporating over 3,000 US counties.

This combination of all four aspects clearly distinguishes this paper from the rest of the existing literature and emphasizes its unique contribution. In particular, the research differs from research conducted in related fields by Alesina et al. (2016), Ager and Brückner (2013a, 2013b), and Sequeira et al. (2018).

Alesina et al. (2016) look at the relationship of diversity and economic prosperity. Their timeframe is 10 years, evaluating a 120 country-sample using a short panel with data for 1990 and 2000. They propose a new index of population diversity and focus especially on the impact of the migrants' skill-level and the prosperity level of the receiving as well as sending country. Neither the long-term impact of diversity on a decade by decade basis, nor the potentially negative impact of diversity with regards to the social tension literature, nor the subnational level or internal migrants are analysed in this paper.

Ager and Brückner (2013a) analyse the effects of changes in population diversity on output growth. They employ 1870–1920 as their period of analysis, which corresponds to the base years of analysis employed in our manuscript. They follow a within-county estimation approach of the US county sample across the fifty-year time frame using a first difference estimation. Despite the fact that Ager and Brückner also refer to diversity as two-dimensional (made up of the two components fractionalization and polarization), they do not extend their analysis beyond the 50-year historical period to the present day economic development and do not evaluate the dynamic impact of diversity on a decade by decade basis.

Ager and Brückner (2013b) is the closest to the aim of the paper. As in our case, they consider the dynamic character of the impact of migrant diversity (measured in 1870, relative to our measurements in 1880 and 1910) on economic prosperity in recent decades. However, there are also substantial differences in the two approaches. First, Ager and Brückner (2013b) define diversity as genetic diversity based on a migratory-distance, using predicted genetic diversity index as suggested by

Ashraf and Galor (2013). Therefore, in contrast to our approach, they define diversity based on biology, genes and ancestry rather than including the two frequently discussed proxies of fractionalization and polarization which our paper refers to. Second, their analysis uses a raft of historical controls for 1870 or even for the period between 1870 and 1920, but does not take into consideration more recent factors that play an important role in current county levels of development. They rely, instead, on state fixed-effects (FE) or a combination of state and county FEs and tend not to report the coefficients of the controls. Third, Ager and Brückner (2013b) only consider external migration and not both internal and external migration in their analysis. Finally, they aim to address potential endogeneity problems by using a combination of Ashraf and Galor's (2013) measure of countries' genetic diversity and controlling for initial GDP per capita. Our research also includes a raft of lagged variables, comprising, among others, initial GDP per capita. But we consider that, given the long-term dimension of the analysis, this is not sufficient to fully address endogeneity issues. This is why we develop a fully-fledged Instrumental Variable (IV) analysis in the manuscript.

Finally, Sequeira et al. (2018) cover the exact same question as Rodríguez-Pose and von Berlepsch (2014). They estimate the effect of migration during the Age of Mass Migration on economic outcomes approximately 100 years later. Their model connects the historical shares of foreign-born migrants in a county (from 1860-1920) to levels of economic prosperity, measured by income, poverty, employment, urbanization and educational attainment, in the 21$^{st}$ century. Their research does not take into account population diversity at county level, nor the cultural composition of the migrant population in historical times.

In brief and to the extent of our knowledge, there is no other piece of research that deals with the long-term impact of population diversity on a decade by decade basis for a period of 130 years (1880-2010), a) considering both internal and external migration; b) controlling for both factors that may have determined diversity more than a century ago and current levels of development; and c) carefully taking into account issues on endogeneity. In this respect, the manuscript makes a substantially novel contribution to our understanding of how population diversity affects economic performance in the very long-term, a topic that has been mostly overlooked by the literature.

## Section 2

***Measuring diversity at county level in 1880 and 1910***

Why are both long-distance internal as well as external immigrants included in the calculation of the diversity indexes? The inclusion of both long-distance internal and external migrants is based on a series of preliminary analyses comparing the potential long-term economic impact of different types of internal and international migrants. Tables S1 and S2 display the results of the link between the presence of immigrants in a given country in 1880 and 1910, respectively, and income per capita in that county in 2010. Each OLS regression concentrates on a different type of migrant origin: Column 1 focuses on all immigrants (both international as internal) living in a given county; Column 2 distinguishes between internal and international immigrants; Columns 3 to 5 use the share of international migrants as a control while focusing on different types of American-born population shares. Column 3 depicts the part of the county population born and living in the same state; Column 4 treats those internal migrants within the population composition of a given US county which were born in any neighbouring state; while Column 5 displays the results for long-distance internal migrants.

The results suggest that internal migrants (especially those that travelled over long distances across the US before they settled) have left an important long-term economic imprint, much in the same way as international migrants. Both groups are also significantly different to the local-born population in their long-term economic influence. Long-distance internal migrants, just as their international counterparts, have left a distinctive trace for long-term economic development which persists more than 100 years after the initial influx of migrants.

Consequently, Tables S1 and S2 provide a first insight into the potential complementarity of internal and international migrants, which should be taken into account when considering population diversity.

By contrast, within-state internal migrants and those stemming from neighbouring states are not included in the measurement of diversity, as it can be argued that their contribution to diversity and their economic legacy are more limited (as indicated by the coefficients in Tables S1 and S2). This discards the great majority of migrants – in 1880 80% of all internal migrants settled less than 500 km from their place of birth – and concentrates only on those that travelled larger distances and were, therefore, more diverse. Short-distance internal migrants are consequently equated with local population.

**Table S1**
**Analysis of the long-term impact of internal migration on economic development (OLS 1880)**

| Dep. Var.: income per capita 2010 (ln) | (1) | (2) | (3) | (4) | (5) |
|---|---|---|---|---|---|
| *All migrants 1880* | 0.140*** | | | | |
| | (0.0249) | | | | |
| *Internal migrants 1880* | | 0.0611** | | | |
| | | (0.0280) | | | |
| *Origin same state 1880* | | | -0.0611** | | |
| | | | (0.0280) | | |
| *Origin neighbour state 1880* | | | | -0.0505 | |
| | | | | (0.0362) | |
| *Origin rest of country 1880* | | | | | 0.135*** |
| | | | | | (0.0384) |
| *International migrants 1880* | | 0.263*** | 0.263*** | 0.241*** | 0.280*** |
| | | (0.0400) | (0.0400) | (0.0399) | (0.0398) |
| Controls for 1880 | YES | YES | YES | YES | YES |
| Controls for 2000 | YES | YES | YES | YES | YES |
| Observations | 2,440 | 2,440 | 2,440 | 2,440 | 2,440 |
| State fixed effects | YES | YES | YES | YES | YES |
| $R^2$ | 0.682 | 0.686 | 0.686 | 0.686 | 0.688 |

Robust standard errors in parentheses, clustered at the state level.
*** p<0.01, ** p<0.05, * p<0.1

**Table S2**
**Analysis of the long-term impact of internal migration on economic development (OLS 1910)**

| Dep. Var.: income per capita 2010 (ln) | (1) | (2) | (3) | (4) | (5) |
|---|---|---|---|---|---|
| *All migrants 1910* | 0.0717*** | | | | |
| | (0.0211) | | | | |
| *Internal migrants 1910* | | 0.0376* | | | |
| | | (0.0225) | | | |
| *Origin same state 1910* | | | -0.0376* | | |
| | | | (0.0225) | | |
| *Origin neighbour state 1910* | | | | -0.0147 | |
| | | | | (0.0336) | |
| *Origin rest of country 1910* | | | | | 0.0740** |
| | | | | | (0.0316) |
| *International migrants 1910* | | 0.198*** | 0.198*** | 0.188*** | 0.203*** |
| | | (0.0492) | (0.0492) | (0.0493) | (0.0492) |
| Controls for 1910 | YES | YES | YES | YES | YES |
| Controls for 2000 | YES | YES | YES | YES | YES |
| Observations | 2,617 | 2,617 | 2,617 | 2,617 | 2,617 |
| State fixed effects | YES | YES | YES | YES | YES |
| $R^2$ | 0.673 | 0.674 | 0.674 | 0.674 | 0.675 |

Robust standard errors in parentheses, clustered at the state level.
*** p<0.01, ** p<0.05, * p<0

## Section 3

### *Introducing fractionalization and polarization indices together*

Figure 4 may suggest that regression models with a quadratic in the fractionalization index, but excluding the polarization index, could have very similar predictive power to the results presented in the paper with a linear effect of fractionalization combined with a linear effect of polarization. However, including the linear combination of fractionalization and polarization is closely related with the research design of the paper. By including fractionalization and polarization independently, we aim at identifying the individual effects of both variables on economic development. If we were to incorporate only one index at a time, an important aspect of diversity impacting economic growth in our model would be missed: a lack of i.e. the polarization aspect of diversity in the regression analysis would result in a transfer of its effect onto the fractionalization index. The estimates would therefore not capture independent effects, but suffer from omitted variable bias.

Moreover, the choice of a linear combination of both complementary indices of diversity follows the relevant literature, such as Montalvo and Reynal-Querol (2005a, b) or Ager and Brückner (2013a). Plotting both indices in our data sample delivers an almost carbon-copy of scatterplots of both variables retrieved in the aforementioned works, as well as in Gören (2014) and Bove and Elia (2017).

Running the regression replacing the polarization index by the quadratic fractionalization index, the impact of diversity is completely eliminated and all significance levels lost (see Table S3). However, if we estimate the same model but drop the quadratic term, we retrieve again the strongly significant results reported in our main variable of interest (see Table S4). Control variables are in both model specifications very similar to the original coefficients, just as the $R^2$ measures.

Similar results are obtained when running the time shift regressions. The quadratic term eliminates the effect of diversity, but the linear combination does not. This indicates that the quadratic term cannibalizes the effect of diversity on income per capita. We therefore prefer, following Montalvo and Reynal-Querol (2005a, b) or Ager and Brückner (2013a), including the linear combination of both indices.

**Table S3**

**Analysis of the effect of diversity on economic development – Replacement of polarization index by squared fractionalization index**

| Dep. variable: Income p.c. 2010 (ln) | (1) 1880 OLS | (2) 1900 OLS | (3) 1910 OLS |
|---|---|---|---|
| *Fractionalization ~* | 0.0155 | 0.0346 | 0.0679 |
| | (0.101) | (0.0860) | (0.0734) |
| *Fractionalization squared~* | 0.123 | 0.133 | 0.0799 |
| | (0.109) | (0.0877) | (0.0717) |
| *Education 2000* | 0.0125*** | 0.0125*** | 0.0123*** |
| | (0.000782) | (0.000828) | (0.000822) |
| *Total population 2000 (ln)* | 0.00158 | 0.00289 | -0.00257 |
| | (0.00566) | (0.00559) | (0.00562) |
| *Share of black population 2000* | -0.00135*** | -0.000914* | -0.00142*** |
| | (0.000456) | (0.000483) | (0.000488) |
| *Female participation 2000* | -0.000157 | 0.000231 | 0.000568 |
| | (0.00117) | (0.00110) | (0.00107) |
| *Unemployment 2000* | -0.0247*** | -0.0265*** | -0.0247*** |
| | (0.00465) | (0.00422) | (0.00455) |
| *Infant mortality 2000* | -6.88e-05 | -0.000160 | -0.000124 |
| | (0.000326) | (0.000290) | (0.000287) |
| *Agriculture 2000* | -0.000271 | -0.000360 | -0.000325 |
| | (0.00207) | (0.00223) | (0.00227) |
| *Mean income (ln)~* | -0.000694 | -0.000541 | -0.00535* |
| | (0.00335) | (0.00407) | (0.00287) |
| *Literacy~* | 0.0995** | 0.0449 | 0.0715 |
| | (0.0389) | (0.0879) | (0.0513) |
| *Total population~ (ln)* | -0.0119** | -0.0120 | -0.00745 |
| | (0.00484) | (0.00744) | (0.00803) |
| *Share of black population~* | 0.220*** | 0.174*** | 0.210*** |
| | (0.0445) | (0.0376) | (0.0426) |
| *Female participation~* | 0.0347 | -0.0309 | 0.000164 |
| | (0.0894) | (0.0895) | (0.0513) |
| *Unemployment ~* | -0.00894 | -0.0466** | -0.208 |
| | (0.00931) | (0.0190) | (0.163) |
| *Agriculture~* | -0.0713 | -0.000247 | -0.0960*** |
| | (0.0538) | (0.0660) | (0.0202) |
| State controls | Yes | Yes | Yes |
| Observations | 2,825 | 3,024 | 3,094 |
| R-squared | 0.642 | 0.636 | 0.641 |

~ Variables date from respective year of migration 1880, 1900, or 1910
Note: Robust standard errors in parentheses, clustered at the state level.
*** p<0.01, ** p<0.05, * p<0.1

**Table S4**

**Analysis of the effect of diversity on economic development – Fractionalization index only**

| Dep. variable: Income p.c. 2010 (ln) | (1)<br>1880 OLS | (2)<br>1900 OLS | (3)<br>1910 OLS |
|---|---|---|---|
| *Fractionalization ~* | 0.123*** | 0.141*** | 0.133*** |
| | (0.0449) | (0.0414) | (0.0309) |
| *Education 2000* | 0.0125*** | 0.0124*** | 0.0122*** |
| | (0.000786) | (0.000829) | (0.000820) |
| *Total population 2000 (ln)* | 0.00106 | 0.00322 | -0.00228 |
| | (0.00574) | (0.00577) | (0.00574) |
| *Share of black population 2000* | -0.00131*** | -0.000904* | -0.00140*** |
| | (0.000464) | (0.000499) | (0.000504) |
| *Female participation 2000* | -0.000115 | 0.000222 | 0.000544 |
| | (0.00116) | (0.00111) | (0.00108) |
| *Unemployment 2000* | -0.0251*** | -0.0264*** | -0.0246*** |
| | (0.00468) | (0.00429) | (0.00458) |
| *Infant mortality 2000* | -7.61e-05 | -0.000149 | -0.000115 |
| | (0.000325) | (0.000288) | (0.000287) |
| *Agriculture 2000* | -0.000349 | -0.000324 | -0.000209 |
| | (0.00208) | (0.00231) | (0.00229) |
| *Mean income (ln)~* | -0.000206 | 0.000553 | -0.00507* |
| | (0.00332) | (0.00404) | (0.00291) |
| *Literacy~* | 0.104*** | 0.0417 | 0.0760 |
| | (0.0375) | (0.0895) | (0.0521) |
| *Total population~* | -0.0113** | -0.0125 | -0.00777 |
| | (0.00482) | (0.00760) | (0.00823) |
| *Share of black population~* | 0.225*** | 0.176*** | 0.212*** |
| | (0.0451) | (0.0381) | (0.0431) |
| *Female participation~* | 0.0213 | -0.0353 | -0.00178 |
| | (0.0915) | (0.0899) | (0.0510) |
| *Unemployment ~* | -0.00993 | -0.0454** | -0.202 |
| | (0.00894) | (0.0192) | (0.164) |
| *Agriculture~* | -0.0667 | 0.0106 | -0.0942*** |
| | (0.0532) | (0.0649) | (0.0202) |
| *State controls* | *Yes* | *yes* | *Yes* |
| Observations | 2,825 | 3,024 | 3,094 |
| R-squared | 0.642 | 0.636 | 0.641 |

~ Variables date from respective year of migration 1880, 1900, or 1910

Note: Robust standard errors in parentheses, clustered at the state level.

*** p<0.01, ** p<0.05, * p<0.1

**Section 4**

***The spatial nature of the data***

In any type of analysis involving geographical units, inter-territorial spatial spillovers could be at play and affect the econometric estimations. Differences in population diversity in surrounding areas may have affected the long-term economic performance of any given county in the US. Although, from a theoretical perspective, there are no clear mechanisms through which the presence of variations in diversity in neighbouring areas more than 100 years ago may continue to influence levels of GDP per head in US counties today, we have assessed whether there is any evidence of the enduring effect of spatial spillovers by calculating Moran's I in conjunction with Local Indicators of Spatial Association (LISA) maps (Figures S1 and S2).

**Figure S1**

**Moran's scatterplots of migration data for queen-1 weight (base year 1880)**

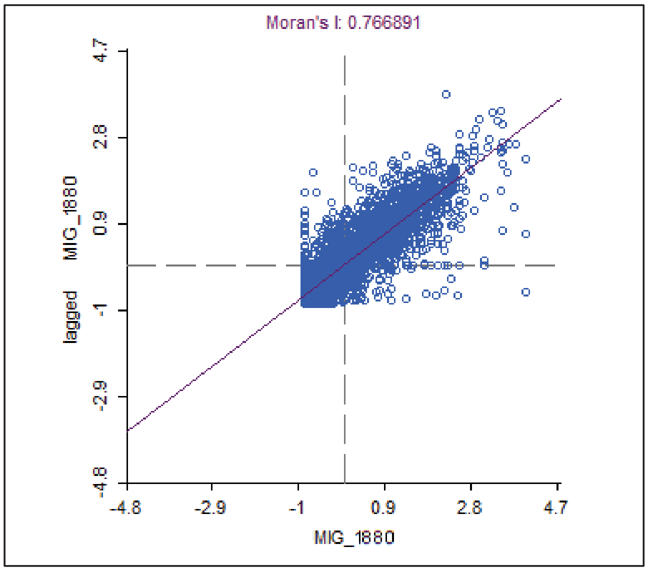


Source: Own elaboration using data from Ruggles, et al. (2015)

**Figure S2**

**LISA significance and cluster maps for queen-1 weight**

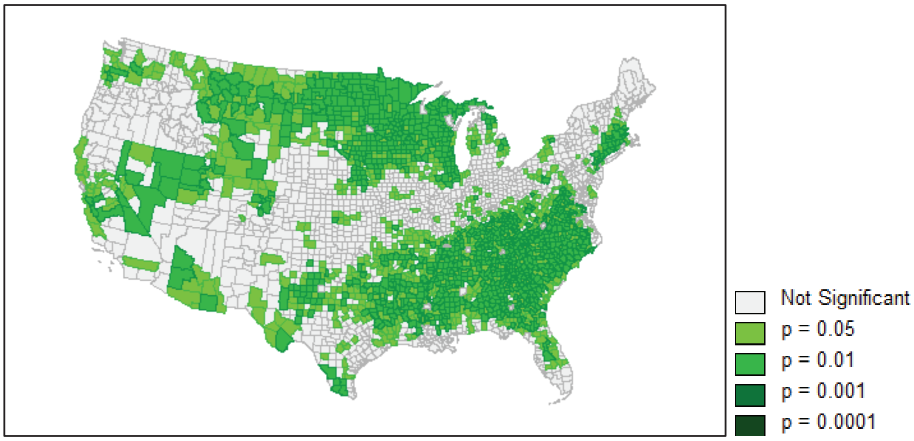


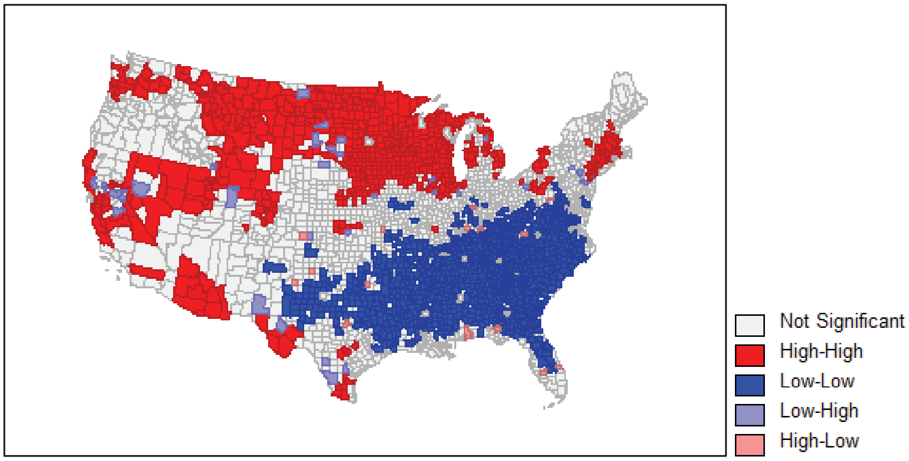


Source: Own elaboration using data from Ruggles et al. (2015)

Both figures point to the presence of spatial patterns in the migration data. Figure S1 displays the Moran's scatterplot for a queen-1 weight, indicating the Moran's I at the top, while Figure S2 displays the equivalent LISA significance and cluster maps for the migration data. Both the Moran's I as well as the LISA maps indicate a non-random distribution of migrants across space. Most observations lie in the upper right quadrant underlining the path dependence of migration. High migration regions seemingly attracted more arrivals developing regional clusters. Thus, high migration counties are close to other counties that also acted as magnets for migrants. Similarly, low migration receiving counties (i.e. the South) are close to equally low endowed ones. This is again displayed in the LISA maps (Figure S2).

There are several options to deal with spatial dependence. One potential option would be to include spatially lagged variables in the model in order to control for the regional spill-overs of migration. This would address the risk of biased estimators due to omitted variables and the consequential endogeneity problems. However, spatial econometric models are not always a straightforward solution as they have potential drawbacks. Gibbons and Overman (2012) and Gibbons et al. (2015) refer, for example (in the case of a model with a spatially lagged independent variable), to (1) sorting problems which may result in an endogeneity bias caused by correlations between the spatially-lagged variable and the neighbouring regions' error term; (2) common shocks which trigger the presence of correlated unobservables which, in turn, result in endogeneity and therefore biased estimators; or (3) the standard omitted variables problem causing biased results. Moreover, researchers that have embarked on using spatially lagged variables to assess long-term growth in US counties have found the spillover coefficients to be imprecisely estimated and not statistically different from zero (Sequeira et al., 2018: 29), meaning an improbable presence of spatial spillovers from population diversity after more than a century.

A second option to address the potential spatial endogeneity issues which could distort the OLS results involves alternative identification strategies, such as fixed or random effects models with panel data, instrumental variable techniques, or experiments/quasi-experiments. These identification strategies deal with the threats to internal validity caused by endogeneity/omitted variables problems and sorting issues in a different way than spatial econometric models. In the paper, we have decided to take one of these alternative routes and resort to the use of instrumental variable (IV) estimation. The IV estimator provides a way to obtain consistent parameter estimates as it eliminates the bias when omitted variables cause biased estimates (in other words, when the unobservables are correlated with the independent variables of interest). Using an instrumental variable which captures the exogenous variation in the variable of interest, IV estimation strategies are designed to isolate the part of the independent variable of interest which is uncorrelated with the error term. This part is then used to estimate the effect of a change in the (in our case) diversity index on income.

By using IV estimation techniques, rather than spatial econometric methods, we align ourselves with much of the relevant literature in this field (e.g. Ager and Brückner, 2013a; Alesina et al., 2016; Bove and Elia, 2017).
